# Neural correlates of reduced sensitivity to information about uncertainty during valuation in older adults: An fNIRS study

**DOI:** 10.1162/IMAG.a.61

**Published:** 2025-06-24

**Authors:** Xue-Rui Peng, Indra Bundil, Stefan Schulreich, Shu-Chen Li

**Affiliations:** Chair of Lifespan Developmental Neuroscience, Faculty of Psychology, Technische Universität Dresden, Dresden, Germany; Centre for Tactile Internet with Human-in-the-Loop, Technische Universität Dresden, Dresden, Germany; International Max Planck Research School on Cognitive NeuroImaging (IMPRS CoNI), Max Planck Institute for Human Cognitive and Brain Sciences, Leipzig, Germany; Cardiff University Brain Research Imaging Centre, School of Psychology, Cardiff University, Cardiff, United Kingdom; Nutrition and Decision Neuroscience Laboratory, Department of Nutritional Sciences, Faculty of Life Sciences, University of Vienna, Vienna, Austria; Vienna Cognitive Science Hub, University of Vienna, Vienna, Austria

**Keywords:** uncertainty reduction, decision making, frontoparietal cortex, fNIRS, statistical inference, aging

## Abstract

Being able to update subjective values based on new information in decision contexts with uncertainty is an important aspect of adaptive cognition. By combining the assessment of updating behavior during a gamble bidding task with measuring frontoparietal activity using functional near-infrared spectroscopy (fNIRS) in older adults (66 to 83 years), this study shows preservation but also impairments of updating behavior in old age. In line with previous results observed in younger adults, older adults update values when new information helps to reduce uncertainty; however, they also update values non-normatively in situations when this is not the case. Using a Bayesian model to fit the behavioral data and to conduct a model-based analysis of brain activity indicate that value updating in older adults is mainly driven by the process of expectation violation instead of belief updating. Older adults with greater expectation violation-related brain activity in the left dorsolateral prefrontal cortex (DLPFC) showed greater deviations from normative predictions in their updating behavior. Considering these results together with earlier findings in younger adults as well as results from secondary analyses directly comparing older with younger adults reveals reduced sensitivity to uncertainty reduction in older adults and age-related differences in subprocesses during valuation and their brain correlates.

## 1 Introduction

Making good decisions in dynamic and uncertain environments requires the ability to discern whether and which further information may potentially reduce uncertainty. Previous studies in young adults have shown that people form internal models (also known as beliefs) based on their experiences or the environmental statistics to predict (anticipate) choice or action outcomes. These beliefs can be updated upon receiving new environmental inputs, thereby adaptively assessing and representing uncertainties in the task contexts (Behrens et al., 2007;Itti & Baldi, 2009;Ma & Jazayeri, 2014;Nassar et al., 2010;Payzan-LeNestour & Bossaerts, 2011). Neural activity in the lateral frontoparietal regions has been shown to track such belief updating during various tasks (Gläscher et al., 2010;Kobayashi & Hsu, 2017;Nour et al., 2018;Tomov et al., 2018).

Cognitive aging may compromise the ability to update statistical features in the context of a given decision task at hand, particularly under uncertainty (Hess, 2014;Samanez-Larkin & Knutson, 2015). Indeed, older adults often show performance deficits in tasks that require adaptive updating. They typically tend to use simpler and computationally less demanding strategies compared with younger adults (Bolenz et al., 2019;Eppinger et al., 2015). For instance, older adults are more likely to employ simple heuristics, such as “win-stay-lose-shift,” that is based solely on the outcome of the previous trial (Worthy et al., 2012). Relatedly, during value-based learning, older adults perform comparably with younger adults when learning from immediate choice outcomes; however, they struggle with making use of information based on delayed outcomes which would require learning the complex transition structures of the task (Eppinger et al., 2013,^2015^). Moreover, older adults are less flexible in adapting their decision-making strategies in situations when task structure changes (e.g., changing levels of environmental uncertainty), which has been interpreted as an impairment in forming clear representations of volatile, uncertain decision contexts (Bolenz et al., 2019;Hämmerer et al., 2019;Nassar et al., 2016). Similarly, in a multidimensional probabilistic learning paradigm that requires selective attention, older adults show higher reliance on simple deviation (error) of reward prediction, whereas younger adults use a more computationally demanding Bayesian approach to identify which dimensions of the environment are relevant for action outcomes (Daniel et al., 2020). Furthermore, when rare events occur, older adults tend to overweight these surprising outcomes more than younger adults (Koch et al., 2024).

From a neurocognitive perspective, the aforementioned behavioral differences in older relative to younger adults may result from age-related deteriorations of the frontoparietal cortex (Kennedy et al., 2015;Raz et al., 2005). These regions have been shown to implicate the detection of environmental changes and the updating of existing mental models (i.e., priors) based on new information (Gläscher et al., 2010;Tomov et al., 2018;Waskom et al., 2014). For instance, deficiencies in learning state transitions in choice–outcome associations during sequential decision making in older adults were associated with the under-recruitment of several frontal regions, including the dorsolateral prefrontal cortex (DLPFC) (Eppinger et al., 2015). Furthermore, other studies showed that aging is associated with a reduction in the selectivity of neural responses to different types of information (Koen & Rugg, 2019;Koen et al., 2020). Such neural dedifferentiation and less distinct representations of different environmental signals have been computationally linked to aging-related declines in neuromodulation (Eppinger et al., 2011;Li et al., 2001; seeLi & Rieckmann, 2014for review), which could further affect the processing of information about uncertainty during decision making.

Albeit the evidence reviewed above, most research on aging and value-based decision making has thus far focused on effects of outcome magnitudes or outcome probabilities. In these studies, updating of mental models about the decision contexts and the expected values of different choices would be mainly driven by the differences (e.g., reward prediction error) between expected and actual outcomes (Eppinger et al., 2013;Hämmerer et al., 2011;Samanez-Larkin & Knutson, 2015). The questions about (i) whether older adults are sensitive to different types of information that may or may not be informative about the uncertainty of the decision contexts and (ii) how this ability may be distinguished from the ability to process deviations between expectations and actual outcomes (henceforth expectancy violation) have not been explicitly investigated.

In real life, not all situations involving expectancy violations are informative. Whereas probabilistic events give rise to certain degrees of uncertainty, different situations may involve different types of uncertainty (Ellsberg, 1961). In some circumstances, the probability of a given outcome is known beforehand. Such situations are commonly viewed as*risky*and involve irreducible uncertainty, since there is a certain probability that the anticipated, preferred outcome may not happen, but further information will not reduce this type of uncertainty (e.g.,Fehr-Duda & Epper, 2012;Huettel et al., 2006). However, there are other circumstances in which the probability of anticipated outcomes is unknown, leaving the situation*ambiguous*(e.g.,Tymula et al., 2012). In such cases, the uncertainty could be reduced through gaining further relevant information about the decision context that may help making inferences about the probability of a potential outcome (e.g.,Kobayashi & Hsu, 2017).

Previous studies indicated that younger adults are quite capable to adjust their value updating and belief updating processes depending on the types of uncertainty in the decision contexts. Specifically, they utilize new information differently based on whether it can reduce uncertainty or not (e.g.,Kobayashi & Hsu, 2017;O’Reilly et al., 2013;Peng et al., 2023;Schulreich & Schwabe, 2021). Using a gamble bidding paradigm that included scenarios for dissociating processes of expectancy violation, belief updating, and value updating, neural correlates of these processes in frontoparietal regions were differentiated in younger adults. Specifically, an fMRI study byKobayashi and Hsu (2017)found that during passive viewing of different gamble scenarios, BOLD responses in the bilateral frontal (middle frontal gyrus/superior frontal sulcus) and parietal (intraparietal sulcus) regions correlated with belief updating; activity in prefrontal regions (medial/ventromedial prefrontal cortex) and the left inferior parietal lobule was associated with value updating, and activity in the bilateral anterior insula was correlated with expectancy violation. The fNIRS appears to be a promising tool to measure frontal activity during decision making under uncertainty (e.g.,Li et al., 2019) and other tasks implicating frontoparietal activities of contextual expectations (e.g.,Kang et al., 2022,^2024^). A recent fNIRS study on younger adults further assessed the updating processes in an active variant of the gamble bidding task where hemodynamic responses were assessed when the participants actively evaluated the values of gamble scenarios. Although the precise localizations differed between the studies, possibly due to differences in task details and measurement modalities (fMRI vs. fNIRS), this study identified oxyhemoglobin responses in different frontoparietal channels that were uniquely associated with each of these processes (Peng et al., 2023). However, whether older adults are sensitive to uncertainty reduction and what may be the neural correlates of individual differences in this ability in older age remain unknown.

In the present study, we, therefore, aim to investigate whether older adults’ value updating behavior would be sensitive to the reducibility of uncertainty and the underlying neurocognitive processes. To this end, we acquired behavioral and hemodynamic responses in the frontoparietal regions using fNIRS in a sample of older adults while they performed a modified gamble bidding task (Peng et al., 2023). Given prior findings of less distinctive task representations and the use of simpler strategies in older adults (e.g.,Bolenz et al., 2019;Eppinger et al., 2013;Hämmerer et al., 2019;Nassar et al., 2016), we hypothesized that older adults would show reduced sensitivity to different types of uncertainty (reducible vs. non-reducible) compared with younger adults. Their updating behavior was expected to be more associated with expectancy violation than updating beliefs about the statistical properties of the environment or values of the gambles. As for the neural processes, we expected that Bayesian model-based brain activities (cf.Kobayashi & Hsu, 2017) associated with expectancy violation, belief updating, and value updating to be less distinct in older adults. Since we hypothesized that older adults’ behavior may primarily be associated with the process of expectancy violation, we expected brain–behavior relations mainly in regions showing expectancy violation-related fNIRS responses in older adults.

Moreover, logical reasoning, a key component of fluid intelligence, has been shown to correlate with optimal choice behavior (Eppinger et al., 2015) and updating performance in young adults (Peng et al., 2023). While the ability of logical reasoning typically declines during aging, there are also substantial individual differences in this ability among older adults (Harada et al., 2013). We thus also explored the relationship between logical reasoning and updating performance in older adults. Specifically, we expected that older adults with higher levels of reasoning ability would deviate less from Bayesian model predictions in their updating behavior. Furthermore, to directly examine age-related differences in subprocesses of updating behavior and associated brain correlates, we conducted secondary analyses incorporating data of younger adults from a previously published study using the exact same task and measurement procedure (Peng et al., 2023).

## 2 Method

### 2.1 Participants

Fifty right-handed older adults participated in this study. We focused on the healthy old adult population and screened participants for the following eligibility criteria: normal or corrected-to-normal vision, no history of neurological or psychiatric disorders, no history of substance abuse, no severe physical illness or disability, no medical scalp conditions that might affect fNIRS setup, and right handedness. The study was approved by the local ethics committee of Technische Universität Dresden (approval number: SR-EK-6012021) and was conducted in accordance with the requirements of the local ethical committee. Before starting the experiment, the participants provided their informed consents. Data of four older adults were excluded from the final sample due to low quality or outlier of data (i.e., three due to poor fNIRS signal quality and one due to extreme*β*values in the fNIRS regression analyses; seeSection 2.5.2for details). Thus, the final sample consisted of 46 older adults (age ranged from 66 to 83 years; mean age ± SD: 71.46 ± 3.54 years; 28 females). Sample size was determined through power analysis using G*Power. Based on medium to large effects (*f*= 0.25; cf.Peng et al., 2023),*α*= 0.05, and statistical power of 0.90, our analysis indicated that 43 participants would be sufficient for detecting within-group effects in repeated-measures ANOVAs. We also conducted further power analyses for the secondary age comparisons, with*α*= 0.05, statistical power of 0.90, and small to medium effects (*f*= 0.15), which indicated that 41 participants per age group would be sufficient for detecting age-related effects in 3-way mixed-effect ANOVAs (with 1 between-group factor with 2 levels and 2 within-subject factors with 2 levels each, that is, age group × normativity × valence).

To characterize our healthy older adult sample and for the purpose of secondary analyses of age differences, we also assessed several cognitive covariates. Participants’ verbal knowledge was assessed using the Spot-the-Word Test (Baddeley et al., 1993), while information processing speed was measured with the Identical-Pictures Test (Lindenberger & Baltes, 1997). The two measures are commonly used to describe sample characteristics in lifespan research (e.g.,Li et al., 2004). Furthermore, the Raven’s Progressive Matrices test (Raven et al., 1998) was administered to measure reasoning ability, because this measure has been shown to correlate with optimal choice behavior (Eppinger et al., 2015) and updating performance in young adults (Peng et al., 2023). Due to invalid Raven’s scores (multiple answer selections or excessive non-responses), two participants were excluded from correlational and covariational analyses involving this measure. These cognitive measures were assessed after the gamble bidding task was completed and the fNIRS cap was removed.

For the purpose of direct age comparisons, we conducted secondary analyses that combined data from the current older adult sample and a younger adult sample previously studied inPeng et al. (2023). The younger adult sample consisted of 45 right-handed younger adults aged 18 to 30 years (mean age ± SD: 22.04 ± 2.8 years; 27 females). Both younger and older participants were compensated with 10 Euros per hour for their participation and could receive an additional potential bonus of up to 10 Euros during the resolution draw after the gamble bidding task. Details about the sample characteristics and performance in the cognitive covariate tasks of both age groups are presented inTable 1.

**Table 1.** Sample characteristics and performance in the cognitive tests by younger and older adults.

	YA *M (SD)*	OA *M (SD)*	YA vs. OA *t (p)*
*N*	45	46	-
Age	22.04 (2.80)	71.46 (3.54)	-73.92 (<0.001)
Gender	18M / 27F	18M / 28F	0.00 ^a^ (1)
Raven Raw Score	53.07 (4.17)	44.61 (5.81)	7.87 (<0.001)
Identical-Pictures Test			
Score	34.51 (4.40)	22.63 (3.18)	14.46 (<0.001)
RT [ms]	2051.37 (300.81)	3221.81 (494.95)	-13.66 (<0.001)
Spot-the-Word Test			
Accuracy [%]	65.32 (10.33)	80.58 (6.54)	-8.40 (<0.001)
RT [ms]	4358.90 (1645.45)	5032.23 (1419.89)	-2.09 (0.04)

*Note.*^a^χ^2^value. OA, older adults; YA, younger adults (data of YA are based on a sample studied inPeng et al., 2023); M, mean; SD, standard deviation. Younger adults exhibited better logical reasoning (higher Raven Raw Score) and processing speed (better performance in Identical-Pictures Test Score), whereas older adults showed better verbal knowledge (better performance in Spot-the-Word Test). These age differences aligned well with existing aging findings (Li et al., 2004).

### 2.2 The gamble bidding task

#### 2.2.1 Task design

The participants performed a modified version of the gamble bidding task (identical toPeng et al., 2023; cf. alsoKobayashi & Hsu, 2017;Schulreich & Schwabe, 2021). The task was derived from the established Ellsberg’s urn problem in economics (Ellsberg, 1961) and entailed two types of uncertainty, that is, risk (irreducible) and ambiguity (reducible). We presented participants with gambles that each showed an urn with two to four colored (red, blue, or yellow) balls inside. Each gamble consisted of (a) “risky” ball(s) that was (were) represented by a single color with a known quantity, and (an) “ambiguous” ball(s), depicted by two half-circles of different colors, for which the total number of such balls was known but the distribution of the two colors was unknown (seeFig. 1A). To prevent color bias, specific color assignments for risky and ambiguous balls were randomized across participants (seeSupplementary Text S1for control analysis showing no effects of random assignment of participant to color schemes). The type of uncertainty (i.e., reducible or not) in the gambles was manipulated by the predetermined winning color for each gamble, which determined whether the winning probability is known (i.e., risky) or unknown (i.e., ambiguous). For example, in a gamble with two single blue “risky” balls and two mixed-colored red/yellow “ambiguous” balls, the winning probability would be fully known (i.e., 50%) if blue is specified as the winning color but would be ambiguous if yellow (or red) is predetermined as the winning color (e.g., inFig. 1A).

**Fig. 1.**
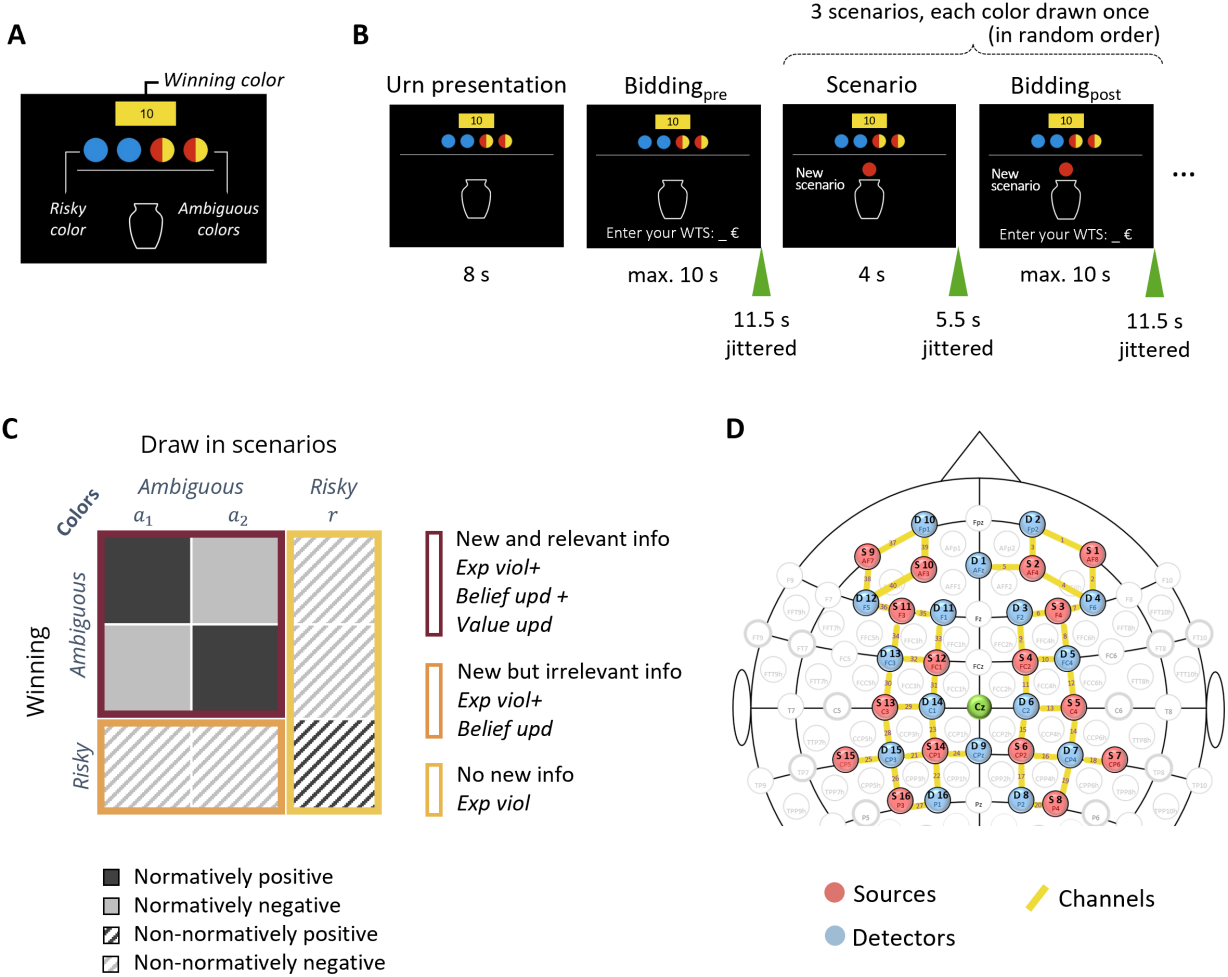
Experimental design and fNIRS montage. (A) Example of an ambiguous gamble with two risky balls (blue) and two ambiguous balls (red/yellow), where the winning color indicated by the color of the small rectangle on top is yellow (adapted fromKobayashi & Hsu, 2017). (B) The trial sequence of an example gamble (adapted fromSchulreich & Schwabe, 2021). (C) Overview of trial categories and information types, illustrating process predictions for different combinations of winning colors and drawn colors in ambiguous and risky gambles. The overview is only shown here for describing the task and was not shown to the participants. (D) Configuration of the frontoparietal fNIRS montage based on the international 10-10 coordinate system, using two 8 × 8 NIRStar systems (40 channels total).

Each gamble started with the presentation of the urn composition. The participants were asked to enter a value to indicate a price in Euro at which they would be willing to sell (WTS) the gamble. All WTS values throughout the experiment were entered as integers from 0 to 10 using a numeric keypad. The WTS value represents the minimum amount of money that a participant would accept to give up their right to potentially win this gamble in a lottery at the end of the task. This is a well-established measure in decision research for assessing subjective valuations (Novemsky & Kahneman, 2005). After the urn content presentation, the participants then observed three distinct scenarios, each representing a hypothetical situation in which one ball is drawn from the urn. Specifically, each scenario involved a draw of one ball, with each of the three colors (red, blue, or yellow) appearing once in a random order across the three scenarios of a gamble (seeSupplementary Text S2for the control analysis showing no potential effects of the draw order). After each scenario presentation, participants again indicated their WTS value using the numerical keypad (seeFig. 1B). As shown inFigure 1C, we manipulated winning colors and corresponding draws to yield different types of information: (1) in ambiguous gambles, scenarios with ambiguous color draws provided new and relevant information that reduces uncertainty about the winning probability of the gambles; (2) in risky gambles, scenarios with ambiguous color draws provided new but irrelevant information, since the winning probability was already known from the initial urn presentation; (3) in both types of gambles, scenarios with risky color draws provided no new information. This task design thus allowed us to evaluate how participants adjusted their subjective valuation of the gamble in response to different types of information (new and relevant, new but irrelevant, or nothing new). Normatively speaking, the WTS values should only be updated if the information obtained from a given scenario is both new and relevant, that is, indicating further information about a potential increase or decrease in winning probability.

Six urn compositions with varying numbers of risky and ambiguous balls were presented (seeSupplementary Table S1for an overview of all urn compositions). The task comprised 24 gambles that were evenly distributed among the 3 possible winning colors: 8 risky gambles and 16 ambiguous gambles (with 8 for each of the 2 ambiguous colors). The order of the gambles was randomized. Each participant provided 24 predraw and 72 postdraw WTS values. To enhance the participants’ motivation and subjective valuations of the gambles, we implemented a final lottery using the Becker–DeGroot–Marschak method (BDM;Becker et al., 1964) for bonus payment. At the end of the experiment, the task program randomly selected one predraw or postdraw gamble scenario for the final resolution draw. Under the BDM procedure, if a participant’s WTS value for the selected scenario was lower than a randomly generated number (between 0 and 10), they would receive this random number as a bonus. However, if the WTS value provided by the participants was higher than the random number generated by the computer, they would play the gamble with a chance to win the maximum of 10 Euros. During the instruction phase, we provided participants with very detailed examples illustrating how different bidding situations under the BDM procedure would affect their potential payoffs, ensuring that older adults comprehended the procedure and could know the bidding situations to maximize their expected payoff (see procedure below,Supplementary Appendixfor the details about our one-on-one interactive task instruction, andSupplementary Table S2for the short quiz used to ensure task comprehension).

#### 2.2.2 Task procedure

Each gamble started with the initial presentation of the urn content composition for 8 s (seeFig. 1B). Afterward, participants were required to enter their predraw bid value (WTS_pre_) within 10 s using the numpad of a standard computer keyboard. If no value was registered within 10 s, the entire gamble was eliminated from the analyses, because in this case the reference value used to calculate the value updating would be missing. Following this, the screen displayed the instruction “Waiting for a new scenario …” on a black background with an average duration of 11.5 s, jittering between 10 and 16 s. The first scenario was then presented for 4 s, followed by the instruction text “Please wait a moment to enter your value…” (the period was jittered between 4 and 10 s, with an average of 5.5 s). Next, participants entered their postdraw bid value (WTS_post_) for the first scenario within 10 s, followed by an inter-scenario interval (mean interval of 11.5 s, jittered between 10 and 16 s). If no value was registered within 10 s, the value updating of the given scenario could not be computed and was considered missing. The same procedure was applied for the second and third scenario of the gamble. Overall, only 0.5% of the trials across all participants were time-outs and excluded from the analyses.

### 2.3 fNIRS data acquisition

The fNIRS data acquisition protocol was the same as in our previous study of younger adults (Peng et al., 2023). To cover the cortex to a larger extent, we employed two NIRSport continuous-wave fNIRS devices (NIRx Medical Technologies, LLC, USA). The two devices were operated in tandem mode using NIRStar acquisition software (version 15.3). Each system was equipped with 8 sources and 8 detectors, with single-tipped optodes, emitting electromagnetic wavelengths of 760 and 850 nm, and sampled at a frequency of 3.472 Hz. The optodes were secured using standard ActiCap grommets (NIRx, Germany), except in the thickly haired parietal regions where spring-loaded grommets (pressure level 2) were used to ensure good scalp contact. Our setup did not include short channels. We maintained a stable channel distance of 3 cm using stabilizing links (NIRx, Germany). The montage included 16 sources and 16 detectors in total, resulting in 40 active channels for data analysis using the placement of optodes based on our previously established frontoparietal montage (as shown inFig. 1D).

The design of this montage was guided by the fNIRS Optodes’ Location Decider (fOLD;Zimeo Morais et al., 2018), aligned with the international 10-10 system and specified by the AAL2 (Automated Anatomical Labeling,Rolls et al., 2015) parcellation. This arrangement effectively covered the bilateral superior frontal gyrus, middle frontal gyrus, superior parietal gyrus, and inferior parietal gyrus, which are areas identified as crucial for belief and value updating processes during the gamble bidding task in younger adults (Kobayashi & Hsu, 2017; for more details on this montage, refer to the previous study byPeng et al., 2023). Proper NIRScap placement was verified according to the international 10-10 location of Cz. An overcap was placed over the NIRScap to block external light and eliminate measurement disturbance.

### 2.4 Procedure

After granting their informed consent, the participants completed a demographic questionnaire and the Edinburgh Handedness Inventory (EHI;Oldfield, 1971). We then measured participants’ head size to select the appropriate fNIRS cap and set up the fNIRS optodes. The built-in calibration and diagnostics feature procedures of NIRStar were run to assess the signal quality of all channels. The software tagged each channel using the labels “excellent,” “acceptable,” “critical,” and “lost.” Before starting the experiment, we repeatedly parted participants’ hair to improve light penetration and repositioned the optodes until at least 35 out of 40 channels displayed excellent signal quality and no “critical” or “lost” channels.

While optodes and caps were being set up, the participants were provided with instructions for the gamble bidding task. To ensure their comprehension, a short quiz of eight separate questions covering three key aspects of the task (i.e., the two types of balls, the independence between scenarios, the bidding procedure, and the rule for winning or losing during the final resolution draw) was administered after the participants had gone through the one-on-one interactive instructions with the experimenter (seeSupplementary Appendixfor the instructions). If participants answered any question incorrectly, the experimenter immediately provided clarification by referring back to the relevant instruction slide before proceeding. Of note, the average accuracy based on the participants’ initial answers for each of the questions in the quiz did not differ between older and younger adults (*t*_89_= -0.73,*p*= 0.46; seeSupplementary Table S2, which also shows results at the level of each single question). Participants then engaged in two practice rounds to familiarize themselves with the task before beginning the main experiment: one round was guided step-by-step by the experimenter, and the other was performed independently. The recording of fNIRS data commenced 30 s before the main task and continued until completion, with the task lasting approximately 38 min on average (ranging from 36 to 42 min).

### 2.5 Data analysis

Behavioral data were analyzed using R (R Development Core Team, 2021). The fNIRS data were analyzed using Matlab R2020b (MathWorks Inc, Natick, MA, USA). The main derived behavioral variables and models used for analyzing the behavioral and fNIRS data are described below.

#### 2.5.1 Behavioral data

In line with previous studies (Kobayashi & Hsu, 2017;Peng et al., 2023;Schulreich & Schwabe, 2021), our primary dependent variable was participants’ value updating, which was quantified as the trial-wise difference between postdraw and predraw values of WTS (∆WTS = WTS_post_– WTS_pre_). The ∆WTSs were categorized based on the normativity and valence of value updating as shown inFigure 1C. In***normatively positive trials***, an ambiguous color draw matches the winning color. In this case, the uncertainty about the gamble is reduced and an increase in winning probability can be inferred, which should normatively result in an upward adjustment of the WTS values. The***normatively negative trials***feature an ambiguous color draw that does not match the winning color in ambiguous gambles. In this case, other than the basic negating feedback, it also suggests a lower probability of winning that should normatively result in a downward adjustment of the WTS values. In other trials, zero value updating was normatively expected because the winning probability remained unchanged even after obtaining further information in the scenarios. However,***non-normative***value updating could still occur based on whether the color-based feedback from a given draw matches (risky color draws in risky gambles) or mismatches (risky color draws in ambiguous gambles and ambiguous color draws in risky gambles) the winning color (Schulreich & Schwabe, 2021). Such updating would rely on a simple negative (or positive) interpretation of the mismatch (or match) between the color drawn and the winning color, possibly driven by expectancy violation, rather than by using uncertainty-reducing information to update the internal model (belief) about the statistical properties of a given gamble (i.e., belief updating).

To investigate to which extent older adults would be sensitive or insensitive to the reducibility of uncertainty in the different gamble scenarios, we analyzed their value updating in each of the aforementioned trial categories. Specifically, we conducted one-sample Wilcoxon signed rank tests (using the*rstatix*::*wilcox_test*function;Kassambara, 2021) to test whether the average ΔWTSs for each trial category differed significantly from zero. The hypothesis was that if older adults are sensitive to uncertainty reduction as the normative Bayesian model predicts, their ΔWTSs should only be significantly above or below zero, respectively, in normatively positive or negative trial categories, but not in any of the three categories of non-normative trial categories.

Biases are often present in human decision-making processes. For instance, a valence-dependent asymmetry in value updating was previously observed in younger adults in normative trials (i.e., information indicating a decrease in winning probability was overweighted relative to information indicating an increase;Peng et al., 2023). We thus also explored whether valence may affect value updating in older adults. To this end, we ran a linear mixed-effect modeling (LMM) analysis, using the*lme4::lmer*function (Bates et al., 2015). Valence, normativity, and their interaction were incorporated as fixed effects to predict the ΔWTSs, whereas the participants were treated as random effects. For a direct comparison, the ΔWTSs in negative trials were sign-flipped to ensure uniform interpretation of increases as greater value updating. Furthermore, the ΔWTSs from the two non-normative mismatch trial categories were merged and averaged before the analysis. The model was estimated using the Restricted Maximum Likelihood (ReML) procedure, and post hoc pairwise comparisons were conducted using the*emmeans*function (Lenth, 2022).

To gain deeper insights into the participants’ updating behavior and underlying processes, we utilized a Bayesian model and compared observed updating behaviors with model predictions (cf.Kobayashi & Hsu, 2017; seeSection 2.5.1.1for details). Predictions of the model provided a quantitative benchmark, against which we evaluated participants’ performance. For the non-normative trial categories, the predicted value updating is zero, which was already integrated in a first set of analysis described above. As for the two normative trial categories, we conducted two one-sample Wilcoxon signed rank tests to examine the deviations of older adults’ΔWTSs from Bayesian model predictions (i.e., deviation (DEV) =ΔWTSs – value updating_predicted_) against 0. The category-wise DEVs were averaged across trials of the same category for each participant. DEVs in normatively negative trials were again sign-flipped for ease of interpretation. Thus, independent of valence, a DEV greater than 0 indicates that the participants’ value updating was larger than the model’s prediction (over-updating), while a negative DEV suggests an under-updating.

According to the Bayesian model, the value updating should be driven by updating beliefs about the winning probabilities associated with the gambles. Nevertheless, expectancy violations, which are inherent in any probabilistic outcomes, may also contribute to non-normative value updating. Here, we quantify this as the probability of a given color ball to be drawn (i.e.,$1-P_{drawcolor}$). To examine the underlying processes of value updating in older adults, we fitted LMMs with predictors of these two distinct processes (i.e., belief updating and expectancy violation) to urn-wise ΔWTSs for each condition (see details of the model onSection 2.5.1.1). Given the secondary aim of also examining age-related differences and to facilitate interpretations, our model selection was guided by analyses conducted on a larger sample including previously published younger adult data (Peng et al., 2023). For the three trial categories with ambiguous draws, we specified an overall model that included age group, belief updating, expectancy violation, and all their interactions as predictors for the observed ΔWTSs. Participants were included as random effects. The backward stepwise model selection process with the*lmerTest::step*function (Kuznetsova et al., 2017) was used for simplifying fixed effects by sequentially testing and removing the least significant terms based on results of likelihood ratio tests. Upon identifying the best fitting model from the full sample (seeSupplementary Table S3), we then adapted it to fit the older adults’ data. For the two trial categories with risky draws, the overall model included age group, expectancy violation, and their interaction as fixed effects, participants as random effects. The subsequent analysis steps for non-normative trial categories were the same as described here for normative trial categories.

To investigate the relationship between individual differences in updating performance and reasoning ability, we conducted Spearman’s correlation between the absolute deviations (|DEVs|) of value updating from Bayesian predictions and raw scores of the Raven’s test (Raven et al., 1998). Furthermore, as a statistical control, we also conducted covariate analysis in the LMMs to examine whether the processes of value updating in older adults would be associated with individual differences in reasoning ability. Specifically, we included Raven’s test scores for each participant as covariates in the models. The main findings remained similar to models without the covariate (seeSupplementary Table S4).

For all LMMs, the*F*-test statistic and*p*-values were based on the Satterthwaite’s method (Kuznetsova et al., 2017). Effect sizes of mixed-effect models (partial eta squared,*η_p_^2^*) were estimated using the*effectsize::eta_squared*function (Ben-Shachar et al., 2020), and interpreted as small (0.01), medium (0.06), and large (0.14) effects. Effect sizes of Wilcoxon tests (rank-biserial correlation,*r_rb_*) were calculated using*rstatix::wilcox_effsize*function (Kassambara, 2021), and interpreted as small (0.1), medium (0.3), and large (0.5) according to the definition byCohen (1988).

##### 2.5.1.1 Bayesian quantitative model

We adopted a Bayesian model (Kobayashi & Hsu, 2017) to predict the updating behavior, which accounts for processes of belief formation and valuation. Formally, belief formation models the probability distribution reflecting the likelihoods of the ball colors in later draws of a given gamble. After the initial urn content presentation, participants have the information about the total number of risky ($n_{r}$) and ambiguous balls ($n_{a})$, but not about the exact distribution of balls in the two ambiguous colors (i.e.,$n_{a1}$and$n_{a2}$). The probability of drawing a risky ball can be simply specified as$P_{pre}(r)$ =$n_{r}\;/\;(n_{r}\;+\;n_{a})$. However, estimating the probability of drawing a ball in one of the two ambiguous color requires considering all possible urn contents, given that the$n_{a1}$and$n_{a2}$are unknown. Assuming a binomial distribution, the probability of$n_{a1}$can be specified as$P_{pre}(n_{a1})\;=\;\frac{1}{2^{n_{a}}}\;\left(\begin{array}{c} n_{a}\\ n_{a1} \end{array}\right)$. Consequently, the probability of drawing a ball in ambiguous colora1can be estimated as$P_{pre}(a1)=\;\sum_{n_{a1}=0}^{n_{a}}P_{pre}(n_{a1})\;\;\cdot \;\;n_{a1}\;/\;(n_{r}\;+\;n_{a}).$During the subsequent scenarios of drawing balls with different colors, when observing a draw in one of the two ambiguous colors (e.g., colora1), the beliefs should be updated under the Bayesian rule, since ambiguity about the color distribution is reduced. In case of a ball in colora1is drawn, the probability of$n_{a1}$would be updated to$P_{post}(n_{a1})\;=\;\frac{1}{2^{n_{a}-1}}\;\left(\begin{array}{c} n_{a}-1\\ n_{a1}-1 \end{array}\right).$In case of a ball in colora2is drawn, the probability of$n_{a1}$is updated to$P_{post}(n_{a1})\;=\;\frac{1}{2^{n_{a}-1}}\;\left(\begin{array}{c} n_{a}-1\\ n_{a1} \end{array}\right).$However, when a ball in the risky color is drawn, the probability of$n_{a1}$remains the same as at predraw because such an event provides no new information about the urn’s content, leaving the degree of uncertainty unchanged. In the model, valuation accounts for the process of computing an expected value (EV) of a given gamble by multiplying the presumed (or beliefs about) probability of the winning color$P_{w}$with the monetary reward of 10€, that is,$EV=10€\times P_{w}$. Because the specific color of a ball being drawn from the urn at any given incidence is probabilistic, each draw can potentially violate expectations. Such expectancy violation at each draw can be quantified as$1-P_{drawcolor}$(seeFig. 1Cfor an overview of scenarios involving expectancy violation, belief updating, value updating, and their combinations as specified by different conditions of the experimental paradigm).

#### 2.5.2 fNIRS data of older adults

##### 2.5.2.1 Data quality check

Before proceeding with data analyses, we evaluated fNIRS signal quality per channel for each participant with HOMER3 toolbox (Huppert et al., 2009). First, the*hmrR_PruneChannels*function was applied to assess the raw light intensities. The settings, including a signal-to-noise ratio threshold of 6.67 and a data range (dRange) between 0.1 to 10, aligned with the ones used in the previous study of younger adults (Peng et al., 2023). Furthermore, we visually checked the power spectral density to identify the heartbeat frequency around 1 Hz. Although such physiological activity is not the focus of our study, the presence of this pattern confirms coupling between the optodes and scalp during the measurement (Hocke et al., 2018;Tong et al., 2011). Channels failing either the automated checks or visual inspections were labeled as low-quality channels. Three participants with more than 13 low-quality channels (over 33% of the 40 channels) were excluded from further analyses (seeSupplementary Table S5for an overview of the number of participants with good signal quality for each of the channels). For the remaining participants, no channels were removed; instead, we employed statistical models that downweigh noisy channels (see details in the next section). This is a conservative approach that may raise type-II error but not false positives (Huppert, 2016;Meidenbauer et al., 2021).

##### 2.5.2.2 Pre-processing pipeline

The NIRS Brain AnalyzIR Toolbox (Santosa et al., 2018) was used to process and analyze the fNIRS data. We applied a pre-processing pipeline that neither involved motion correction nor pre-filtering, but instead used a robust statistical model that is less biased by fNIRS-specific artifacts (Santosa et al., 2018, p. 29). The pre-processing comprised the following steps: the raw data were transformed into optical density and then converted to oxygenated hemoglobin (HbO) and deoxygenated hemoglobin (HbR) by applying the modified Beer–Lambert law (Strangman et al., 2003) that used individual age-related partial pathlength factor (PPF). Specifically, we first calculated the differential path length factor (DPF) for each participant using the equation fromScholkmann and Wolf (2013):$DPF(\lambda ,\text{A})=\alpha \;+\;\beta {\text{A}}^{\gamma }\;+\;\delta \lambda^{3}\;+\;\eta \lambda^{2}\;+\;\zeta \lambda$, whereλis the wavelength and A is age. The other parameters used in the question were also obtained from their empirical data:*α*= 223.3,*β*= 0.05624,*γ*= 0.8493, δ = -5.723 × 10^−7^,*η*= 0.001245, and*ζ*= -0.9025. Next, the DPF was converted into PPF (PPF = 1/60 × DPF).

##### 2.5.2.3 Modeling process-related activations

For the individual level analysis, we employed an autoregressive iteratively reweighted least-squares (AR-IRLS) pre-whitening approach (Barker et al., 2013). Our main model (Model-1) included a regressor with the onsets of the 72 scenario presentations and the related parametric modulators of belief updating, value updating, and expectancy violation of each scenario. Value updating was quantified as the participants’ ΔWTSs, while belief updating and expectancy violation were quantified by values of the Bayesian model’s predictions (seeSection 2.5.1.1above). All modulators were centered and normalized separately. Regressors of no interest included the time triggers for urn presentation and entering bidding values on the keypad at predraw and postdraws. Each regressor was modeled according to its specific duration, which corresponds to either the presentation duration or the response times during the bidding phases. We used the canonical hemodynamic response function (also known as “double gamma function”) with default parameters (peak time 4 s and undershoot time 16 s;Santosa et al., 2018). Prior to the group analysis, we checked the distributions of the*β*values across all participants for each channel. One participant was detected as an outlier (*β*values for 14 channels below 3 SD) and was thus not included in the final sample (*n*= 46).

At the group level, we used LMMs to calculate the group mean for each regressor, where participants were treated as random effects and the other factors of interest were fixed effects. The Student’s*t*-test was performed to calculate and compare the channel-wise regression coefficients. FDR corrections were applied for corrections of multiple comparisons among all data types, channels, and effects of interests. Our rationales for focusing on analyses of HbO signals include their previously observed associations with updating processes (Peng et al., 2023), higher signal-to-noise ratio, and greater sensitivity to task-induced, event-related cortical changes compared with HbR signals (Cheng et al., 2015;Hoge et al., 2005;Huppert et al., 2006;Jiang et al., 2015). Nevertheless, we also conducted analyses of HbR, which showed that no channels yielded significant activity related to any of the updating processes (all uncorrected*p*’s > 0.07). We visualized the group-level HbO results by converting*t*statistic values of channels for which the uncorrected*p*< 0.05 into *.img files using the*nirs2img*function (https://www.alivelearn.net/?p=2230). These images were subsequently overlaid on the 3D brain model using*Surf Ice*(https://www.nitrc.org/projects/surfice/).

Additional analyses were performed to assess the effects of trial category on the average activation during scenario presentations. To investigate the main effects and interactions of normativity and valence, we set up Model-2 which incorporated four regressors of the trial categories: normatively positive, normatively negative, non-normatively positive, and non-normatively negative (seeSupplementary Table S6for the results). To explore whether the valence-dependent HbO activation previously identified in normative trials in younger adults would also be observed in older adults, we set up Model-3, which merged the non-normatively positive and non-normatively negative trials into a single “normatively zero” category (seeSupplementary Table S7for the results). All models incorporated regressors of no interest that were consistent with those in the main model (Model 1) and the group-level model specification followed the same as the main model.

#### 2.5.3 Correlational analysis of value updating performance and HbO responses

We performed correlational analyses to explore the relationship between value updating performance and the HbO responses associated with processes of belief updating and expectancy violation, respectively. For each process, Spearman’s rho was calculated between the*β*values of channels showing significant process-related HbO activation and the absolute deviations (|DEV|) between the observed value updating and the Bayesian model prediction.

#### 2.5.4 Secondary analysis of age differences in value updating

For secondary analyses on adult age-related differences of value updating, we ran a LMM to account for individual and age-related differences in ΔWTSs that included age group (old vs. young), valence (positive vs. negative), normativity (normative vs. non-normative), and their interactions as fixed effects, while participants were treated as random effects.

Since value updating was calculated by subtracting predraw values from postdraw in each trial, the ΔWTSs inherently account for individual differences in subjective values ascribed to the 24 gambles that differed in levels of risk or ambiguity. Nevertheless, it is not known whether there would be age-related differences in predraw values, and if so, whether such differences might potentially contribute to age differences in value updating when compared across studies and samples. To address this, we ran two additional LMMs. The first LMM examined whether predraw values differ between young and older adults and whether these differences differ between gamble types (risky and ambiguous). The model included age group, gamble type, and their interaction as fixed-effect predictors. The averaged predraw WTSs for each participant and gamble type were the dependent variable, with participants included as random effects. The second LMM included age group, gamble type, phase (predraw and postdraw), and their interactions as fixed-effect predictors, with participants WTSs averaged separately for each phase and gamble types as the dependent variable. Participants were treated as random effects. If the three-way (age group × gamble × phase) and two-way (age group × phase) interactions are not significant, it would suggest that the age difference observed in value updating is not due to group differences of subjective values attributed to the gambles at the predraw phase. The models described above were estimated with the ReML approach, and post hoc comparisons were conducted using the*emmeans*function (Lenth, 2022), with*p*-values adjusted by the Holm method.

#### 2.5.5 Secondary analyses of age differences in process-related HbO responses

To explore age differences in underlying neural processes, we combined fNIRS data from the current study of older adults and a previous study of younger adults in secondary analyses. To ensure direct comparability between age groups, younger adults’ fNIRS data were pre-processed using the same pipeline as that used for older adults, including conversion of optical density values to hemoglobin concentrations using the modified Beer–Lambert law with individual age-related PPF. The individual-level analyses for young adults were performed following the identical procedure described above inSection 2.5.2for older adults. We then conducted group-level analyses using LMMs to model the interactions between processes (expectancy violation, belief updating, and value updating) and age groups (younger adults and older adults) on the process-related HbO responses.

## 3 Results

### 3.1 Behavioral results

#### 3.1.1 Older adults showed both normative and non-normative behaviors of value updating

The one-sample Wilcoxon signed rank tests revealed that value updating (ΔWTSs) differed from 0 in all trial categories (i.e., all combinations of normative/non-normative and positive/negative updating, seeSection 2.5.1for detailed descriptions) in the current old-adult sample (seeFig. 2A). Specifically, the value updating was significantly greater than 0 in both normatively positive [*median (Mdn)*= 0.79,*p*< 0.001,*r_rb_*= 0.55] and non-normatively positive trials (*Mdn*= 0.42,*p*= 0.003,*r_rb_*= 0.44). In contrast, it was significantly less than 0 in the normatively negative (*Mdn*= – 1.00,*p*< 0.001,*r_rb_*= 0.75) and non-normatively negative trials, including risky color draws in ambiguous gambles (*Mdn*= -0.46,*p*= 0.003,*r_rb_*= 0.43) and ambiguous color draws in risky gambles (*Mdn*= -0.85,*p*< 0.001,*r_rb_*= 0.72). Significant deviations from 0 in normative trials indicate qualitatively normative updating performance, whereas such deviations in non-normative trials indicate non-normative behavior. For the direct comparison with data from younger adults assessed in a previous study (Peng et al., 2023), see results and plots (Fig. 4A) of secondary analysis inSection 3.4.

**Fig. 2.**
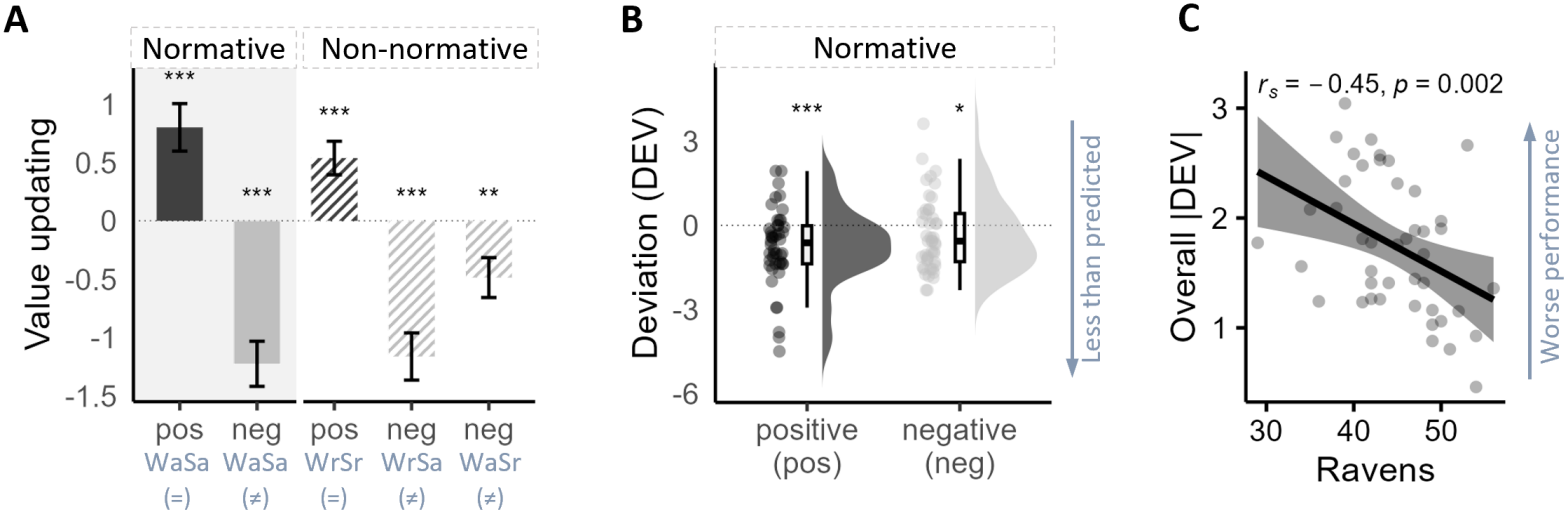
Behavioral results. (A) Value updating in older adults. Trial categories are labeled on x-axis by winning colors (W) and drawn colors in scenarios (S), with “a” standing for ambiguous, “r” for risky, “=“ indicating a match, and “≠” indicating a mismatch between the winning color and drawn color. Error bars show the standard error of the mean. (B) Deviations from Bayesian model predictions in normative trials by valence. Boxplots show the median and quartiles of the data. (C) Correlation between reasoning ability and updating performance. The shaded areas represent the confidence interval of 95%.^***^p< 0.001,^**^p< 0.01, *p< 0.05.

Furthermore, results of the LMM revealed a significant main effect of valence (*F*_135_= 5.87,*p*= 0.02,*η_p_^2^*= 0.04). Post hoc analysis suggested that value updating in positive trials was less than that in negative trials (estimate of difference in marginal means = -0.41,*t*= -2.42,*p*= 0.02). The main effect of normativity was marginally significant (*F*_135_= 3.81,*p*= 0.053,*η_p_^2^*= 0.03) and the interaction of valence × normativity was not significant (*F*_135_= 0.16,*p*= 0.69,*η_p_^2^*= 0.002).

#### 3.1.2 Value updating of older adults deviated from model predictions in normative trials

Even though in normative trials older adults showed qualitatively normative updating performance (ΔWTSs deviated from 0) as the analyses above revealed, their performance can still quantitatively deviate from Bayesian predictions. To further evaluate how much older adults’ updating behaviors deviated from model predictions in normative trials, we analyzed DEVs by taking the difference between observed behavior and model prediction (i.e., DEV = ΔWTS – prediction) for each gamble type (the DEVs of the normatively negative trials were sign flipped for ease of interpretation). The DEVs of value updating were significantly below 0 (normatively positive,*Mdn*= -0.81,*p*< 0.001; normatively negative,*Mdn*= -0.60,*p*= 0.04; seeFig. 2B), showing that the extent of value updating in older adults was less than what the model predicted, which may indicate that older adults underweight new information.

Note that we also observed that older adults’ predraw WTSs were significantly higher than the model’s predictions in both risky and ambiguous gambles (all*p*’s < 0.001). The higher WTSs at predraw than model predictions indicate that, prior to any new information given by observed draws, older adults tend to place higher values to both risky and ambiguous gambles than normatively predicted by the Bayesian model. However, the higher values older adults ascribed to the gambles at the predraw phase did not limit the potential range of values for adjustments at the postdraw phase (seeFig. 4in the section of age comparisons). If such a restriction in range of value updating is present in the data, it would only affect the postdraw WTS in the positive, but not in the negative direction (for which we also observed underweighting of new information). Moreover, results from analyses comparing postdraw WTSs with the model’s predictions in different trial categories showed that the postdraw WTSs were generally still significantly greater than the model’s predictions (*p*’s < 0.001), suggesting an available range for increases above the model predictions, (seeSupplementary Text S3for the details of these analyses).

Together, the results reported in these two sections show that older adults’ updating behaviors were only quasi-optimal in the normative trial categories: they did update the WTS values in these trials, but underweighted new information. Their updating behavior in the non-normative categories deviated completely from the model prediction (i.e., no updating) and showed that they were not sensitive to whether new information can or cannot reduce uncertainty. We also observed that older adults’ value updating was affected by the valence of the new information in both normative and non-normative trials categories. The extent of value updating was larger in trials with a mismatch between draw and winning color (negative trials) than in trials with a match between draw and winning color (positive trials). In addition, prior to any further information given by observed draws, relative to normative predictions, older adults gave the gambles higher values than the Bayesian model (seeSection 3.4for age comparisons).

#### 3.1.3 Individuals with higher Raven’s scores deviate less from model predictions

We further examined the potential relationships between individual differences in basic cognitive abilities reflecting fluid intelligence and value updating performance. We observed a significant negative correlation between Raven’s raw scores and the overall deviations of value updating from Bayesian normative predictions (|DEV|). The result showed that older adults with higher reasoning ability performed better (i.e., deviated less from model predictions) in the gamble bidding task (Fig. 2C). This relationship remained significant even after controlling for individual differences in deviations of predraw values from the Bayesian predictions (semi-partial Spearman correlation*r*= -0.38,*p*= 0.01).

#### 3.1.4 Processes underlying value updating in older adults

As shown inTable 2, results from regression models predicting value updating (ΔWTS as dependent variable) with expectancy violation and belief updating as predictors showed that in normatively positive trials, expectancy violation yielded a significant positive weight in predicting value updating (*β*= 2.03,*p*= 0.036), while belief updating—the process that would be expected to drive value updating from a Bayesian perspective—did not show a significant association. In normatively negative trials, only a statistical trend for belief updating being predictive of value updating in the negative direction (ascribing less values relative to predraw) was observed (*β*= -3.34,*p*= 0.083). In non-normatively positive trials, expectancy violation did not show an effect. In non-normatively negative trials, we found that updating toward more negative values was predicted by expectancy violation for both risky (*β*= -2.90,*p*< 0.001) and ambiguous gambles (*β*= -6.26,*p*< 0.001). Interestingly, belief updating shows a significant negative weight in predicting value updating when observing ambiguous-color draws in risky gambles (*β*= -4.20,*p*= 0.029), for which no updating would be expected from a Bayesian perspective.

**Table 2.** Estimation of linear mixed-effect models on the underlying processes of value updating in different trial categories.

	Coefficients ( *β* s)	SE	DF	95% CI	*p* -value
*Normatively positive (ambiguous-color draws in ambiguous gambles—color matched)*					
(Intercept)	-1.06	0.81	255.09	[-2.65, 0.54]	0.193
Belief updating	1.89	1.88	228.00	[-1.82, 5.59]	0.317
**Expectancy violation**	**2.03**	**0.96**	**228.00**	**[0.13, 3.93]**	**0.036**
*Normatively negative (ambiguous-color draws in ambiguous gambles—color mismatched)*					
(Intercept)	-0.71	0.36	246.43	[-1.43, 0.00]	0.051
Belief updating	-3.34	1.92	229.00	[-7.12, 0.45]	0.083
*Non-normatively positive (risky-color draws in risky gambles)*					
(Intercept)	0.02	0.33	270.68	[-0.64, 0.68]	0.959
Expectancy violation	0.98	0.60	229.49	[-0.20, 2.17]	0.104
*Non-normatively negative (risky-color draws in ambiguous gambles)*					
**(Intercept)**	**3.53**	**1.07**	**243.61**	**[1.42, 5.64]**	**0.001**
**Expectancy violation**	**-6.26**	**1.40**	**228.28**	**[-9.02, -3.50]**	**< 0.001**
*Non-normatively negative (ambiguous-color draws in risky gambles)*					
**(Intercept)**	**1.59**	**0.43**	**273.00**	**[0.75, 2.44]**	**< 0.001**
**Expectancy violation**	**-2.90**	**0.49**	**228.00**	**[-3.87, -1.93]**	**< 0.001**
**Belief updating**	**-4.20**	**1.92**	**228.00**	**[-7.98, -0.41]**	**0.030**

*Note.*Model specification determined by theoretical and empirical considerations (seeSection 2.5.1for details). Value updating (i.e., ∆WTS) in normatively/non-normatively negative trials was not sign-flipped here (i.e., negative coefficients indicate more negative value updating with higher expectancy violation/belief updating). Bold font indicates statistically significant result (*p*< 0.05).

### 3.2 Brain correlates of updating processes

**Fig. 3.**
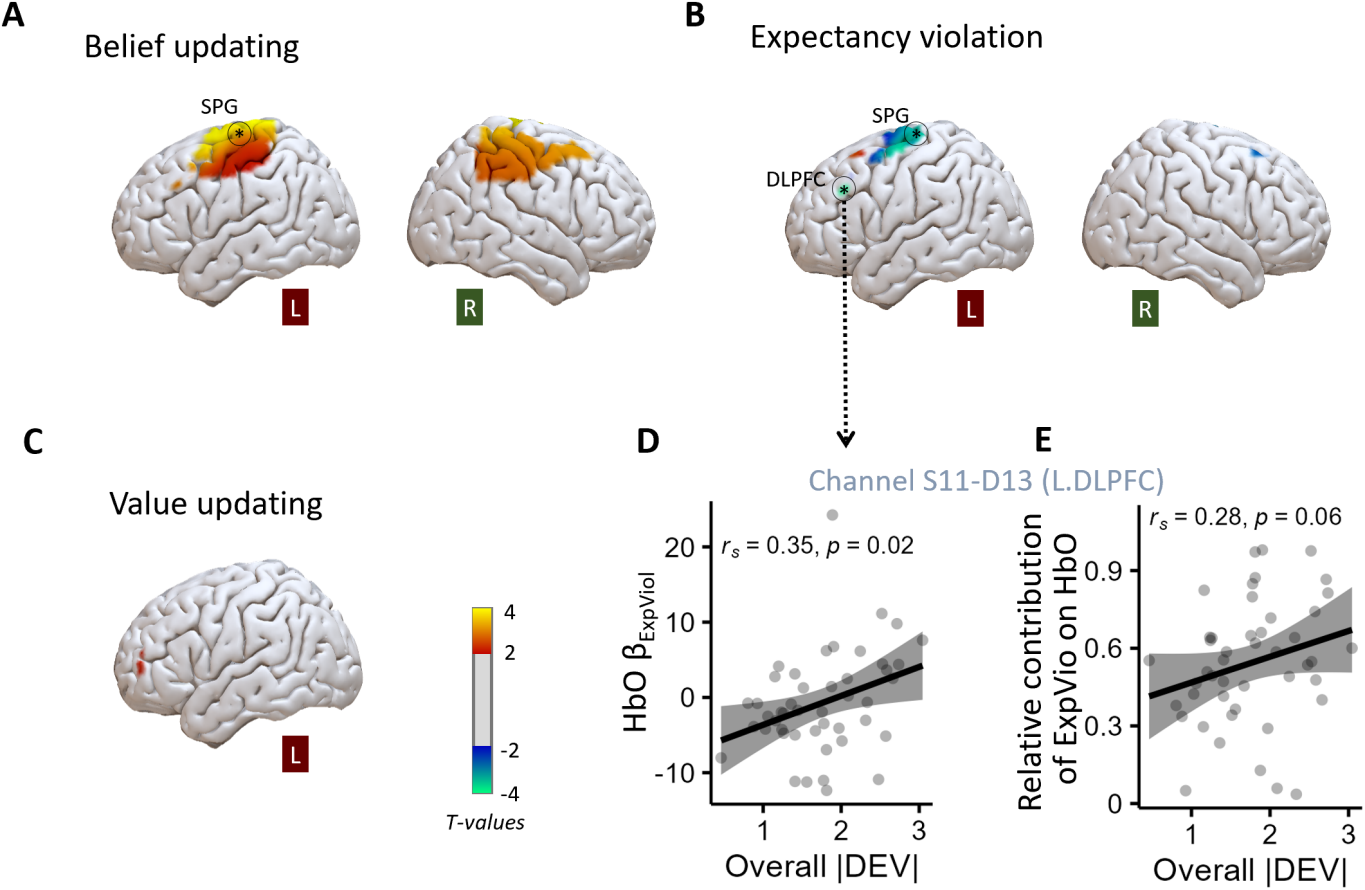
HbO activation in frontoparietal regions correlated with updating processes during observed draws and with task performance in older adults. Neural correlates of (A) belief updating, (B) expectancy violation, and (C) value updating. Activation of channels with uncorrected*p*< 0.05 is shown here. Channels that remain significant after FDR corrections (*q*< 0.05) are indicated with *. SeeSupplementary Table S8for the values of the statistics. (D) Individuals with higher expectancy violation-related HbO activation in the left DLPFC (channel S11-D13) deviated more from the Bayesian model’s prediction. (E) Individuals with greater contribution of expectancy violation (relative to belief updating) in the same channel tend to show larger deviations from model predictions.

The channel-wise parametric analyses of the fNIRS HbO signals revealed a significant positive correlation with belief updating (FDR*q*= 0.002) in the superior parietal gyrus (channel S14-D14;Fig. 3A). In contrast, HbO signals in the SPG (same channel) and in the left DLPFC (channel S11-D13) correlated negatively (FDR*q*’s < 0.05) with expectancy violation (Fig. 3B). No significant correlations between value updating (ΔWTS) and HbO responses were observed (Fig. 3C). For specific statistics values, seeSupplementary Table S8.

### 3.3 Relationships between brain correlates of updating processes and updating behavior

We calculated Spearman correlations between the overall updating performance—quantified by the absolute value of deviations from model prediction (|DEV|)—and the*β*values of the channel in the SPG that showed a significant positive correlation with belief updating (Fig. 3A), and the two channels in SPG and the left DLPFC showing significant negative correlations with expectancy violation (seeFig. 3B). The results revealed that neither the belief updating-related HbO responses in the SPG nor the expectancy violation-related responses in the same channel (S14-D14) were related to updating performance (*p*’s > 0.44). However, as shown inFigure 3D, older adults with higher expectancy violation-related activation in the left DLPFC (channel S11-D13) showed worse overall performance (*r_s_*= 0.35, uncorrected*p*= 0.02, FDR*q*< 0.05). This correlation also remained significant (*r_s_*= 0.32,*p*= 0.027) after controlling for predraw valuation performance and Raven’s scores.

Given that the channel in the left DLPFC (S11-D13) also exhibited some belief updating-related activation at a lenient threshold (*t*= 3.04,*p*= 0.003, FDR*q*= 0.10), we further explored the correlation between overall task performance and the relative influence of belief updating and expectancy violation on HbO activity in this channel. The relative influence was quantified as the absolute standardized*β*value of expectancy violation divided by the sum of the absolute standardized*β*of both predictors, that is, |*β*_expVio_| / (|*β*_expVio_| + |*β*_beliefupd_|). The range of this index is [0, 1], with values closer to 1 indicating a greater influence of expectancy violation on the HbO activation. We found that individuals with a larger relative contribution of expectancy violation in the left DLPFC tended to deviate more from the Bayesian predictions (*r_s_*= 0.28,*p*= 0.06; seeFig. 3E).

### 3.4 Results from secondary analyses of adult age differences

To directly examine effects of aging on value updating, we analyzed an extended dataset that combined data from the current older adult sample with published data of younger adults from a previous study (Peng et al., 2023). We ran an LMM with age group, valence, normativity, and their interactions as predictor variables for predicting ΔWTSs. Other than modeling participants as random effects, the other predictors were modeled as fixed effects. Since Raven’s scores were correlated with value updating performance in both age groups, and the significantly lower scores in older adults compared with younger adults (*p*< 0.001,Table 1), we also included Raven’s scores in the LMM as a control variable. The model revealed no significant main effects of age group (*F*_1,86_= 1.45,*p*= 0.23,*η_p_^2^*= 0.07). However, there were significant main effect of valence (*F*_1,261_= 24.25,*p*< 0.001,*η_p_^2^*= 0.09), normativity (*F*_1,267_= 45.03,*p*< 0.001,*η_p_^2^*= 0.15), as well as a significant 2-way interaction of age × normativity (*F*_1,261_= 12.47,*p*< 0.001,*η_p_^2^*= 0.05). The other interactions were not significant (all*p*’s > 0.31), in particular there was also no age × valence interaction, indicating that although both old and young adults showed stronger value updating for (normatively) negative compared with positive trials, there was no age-related change in this valence bias. As shown inFigure 4A, post hoc tests on the effects of age group on normativity revealed that older adults did not differ from younger adults in the normative trials (OA vs. YA, estimate of difference in marginal means = -0.14,*t*= -0.65,*p*= 0.52), but showed greater non-normative value updating than younger adults in all three categories of non-normative trials (estimate = 0.60,*t*= 2.76,*p*= 0.007). This pattern of results indicates that, unlike younger adults, the updating behavior of older adults was not sensitive to whether new information in the gamble scenarios could or could not reduce uncertainty.

**Fig. 4.**
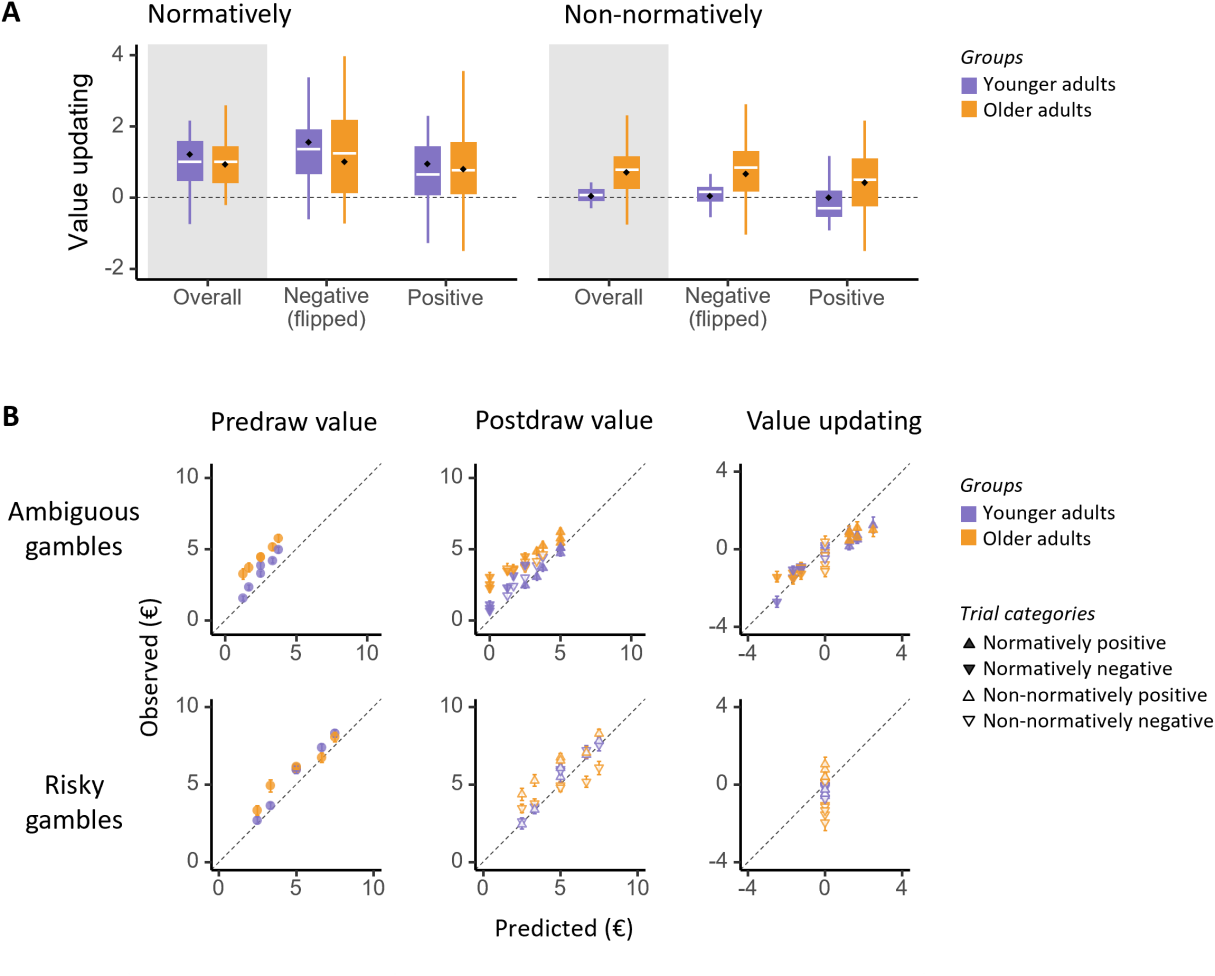
Adult age differences in value updating and subjective values. (A) Value updating in the normative and non-normative trials by age groups (shown here are overall results and separately by valence; signs for negative-valence categories were flipped for comparison). The boxplots display quartiles, with the white horizontal line showing the mean and the black diamond indicating the median. (B) Observed versus Bayesian predicted values shown for predraw (left), postdraw (middle), and value updating (right), separated by ambiguous gambles (top) and risky (bottom) gambles. Each data point represents a specific urn composition. Values of negative-valence categories were not sign-flipped here. Error bars indicate the SE (some not visible due to small size).

Furthermore, we also examined whether younger and older adults differ in the predraw values. We ran an LMM with age group, gamble type, and their interactions as fixed-effect predictors to predict predraw WTSs. Participants were included as random effects, and Raven’s scores were included as a control variable. The model revealed significant main effects of age group (*F*_1,86_= 4.96,*p*= 0.03,*η_p_^2^*= 0.05), gamble type (*F*_1,87_= 162.82,*p*< 0.001,*η_p_^2^*= 0.65), and a significant 2-way interaction of age × gamble type (*F*_1,87_= 11.67,*p*< 0.001,*η_p_^2^*= 0.12). As shown inFigure 4B, post hoc tests on the effects of age group on gamble type revealed that older adults did not differ from younger adults in the predraw values of risky gambles (OA vs. YA, estimate = 0.12,*t*= 0.39,*p*= 0.69), but showed greater predraw values in ambiguous gambles than younger adults (estimate = 1.08,*t*= 3.55,*p*< 0.001).

To further examine whether the subjective values ascribed to gambles of varying risk and ambiguity at the predraw phase might contribute to the observed age differences in value updating, we ran another LMM with age group, gamble type, experimental phase, and their interactions as predictor variables to predict WTSs. Participants were treated as random effects, and Raven’s scores were included as a control variable. As shown inFigure 4B, the model revealed significant main effect of age (*F*_1,86_= 4.53,*p*= 0.04,*η_p_^2^*= 0.05), gamble (*F*_1,261_= 413.52,*p*< 0.001,*η_p_^2^*= 0.61), and phase (*F*_1,261_= 17.19,*p*< 0.001,*η_p_^2^*= 0.06). Although the 2-way interactions of age × gamble (*F*_1,267_= 41.16,*p*< 0.001,*η_p_^2^*= 0.14) were significant, neither the 2-way age × phase (*p*= 0.21) nor the 3-way age × gamble × phase (*p*= 0.40) interactions were significant. Together this pattern of results further indicated that, although older adults subjectively valued the ambiguous gambles more than younger adults at predraw, age group differences in value updating are not due to this effect.

We also conducted secondary analyses to compare age differences in model-based analyses of HbO responses that correlated with processes of expectation violation or updating. First, we conducted channel-of-interest analyses by comparing age differences in channels for which model-based processes correlated with HbO responses in older adults. These analyses showed that in left DLPFC (i.e., channel S11-D13) and in left SPG (i.e., channel S14-D14) where significant negative correlations with expectation violation (i.e., lower values of expectation violation linked to greater HbO responses) were observed in older adults (seeFig. 3Babove), the expectation violation-associated HbO activity was statistically significant in older adults, but not in younger adults (seeFig. 5A). In the left SPG (i.e., channel S14-D14), the belief updating-associated HbO activity did not differ between young and older adults (*p*= 0.11). Furthermore, we also explored age differences across all channels. With a more stringent significance criterion of FDR correction at*q*< 0.05, we found an effect in the left DLPFC (i.e., channel S12-D13) where a significant positive correlation with value updating (i.e., higher values of value updating relating to higher HbO responses) was observed in younger adults (seePeng et al., 2023). The value updating-associated HbO activity was conversely only significant in younger, but not in older adults (seeFig. 5B).

**Fig. 5.**
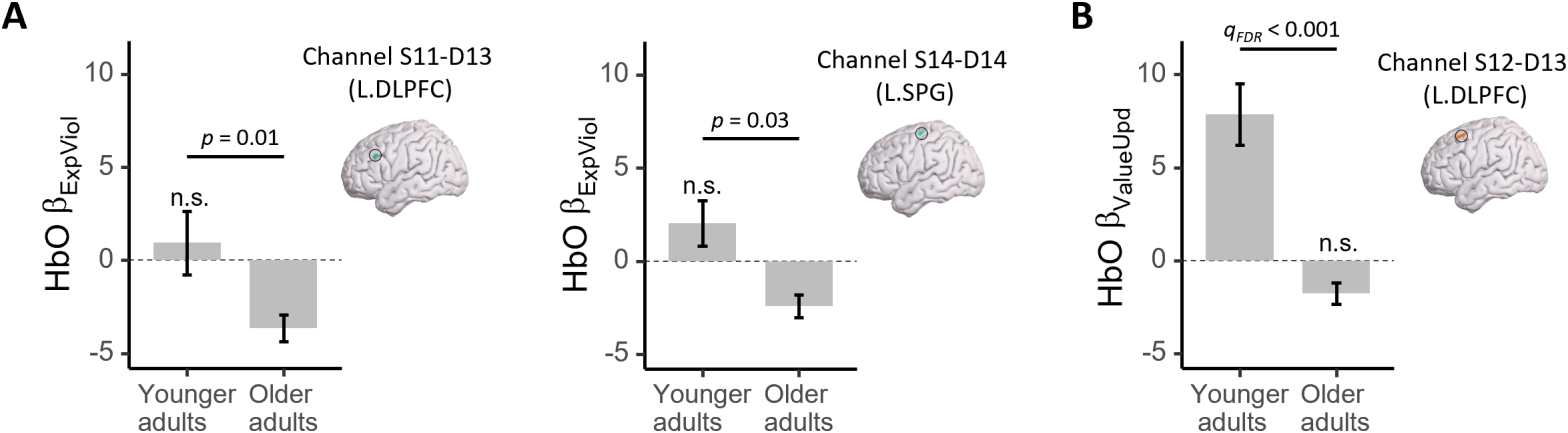
Age-related differences in HbO activation correlated with expectation violation- and updating-related processes. (A) Channel-of-interest analysis in channels for which updating processes significantly correlated with HbO responses in older adults. (B) Analysis over all channels. n.s., not significant.

## 4 Discussion

Extending previous studies on adaptive updating in decision contexts with reducible and irreducible uncertainty, the present study investigated whether older adults’ value-updating behavior would be sensitive to uncertainty reducibility and examined the underlying neurocognitive processes. Using a modified gamble bidding task combined with measuring frontoparietal activities using fNIRS, we were able to dissociate processes of expectancy violation, belief updating, and value updating through formal modeling. Specifically, we tested three main hypotheses: (1) older adults would show reduced sensitivity to different types of uncertainty and their updating behavior would be primarily driven by expectancy violation rather than the change of winning probability; (2) neural correlates of expectancy violation, belief updating, and value updating would be less distinctive in older adults; and (3) older adults with better logical reasoning ability would show updating performance that is more in line with Bayesian model predictions. Our findings largely confirmed these hypotheses. First, older adults updated values after gaining new information, even when the information could not reduce uncertainty about the winning probability of the gambles (Fig. 2A), and their updating behavior was mainly driven by expectancy violation (Table 2). Second, we observed overlapping activity patterns for belief updating and expectancy violation in frontoparietal regions, particularly in the SPG and left DLPFC (Fig. 3;Supplementary Table S8), which suggests less distinct neural representations of these processes in older adults. Third, we found that older adults with better logical reasoning ability (as measured by Raven’s test scores) showed less deviation from the model predictions in value updating (Fig. 2C), suggesting that cognitive abilities continue to play an important role in optimal updating performance even in older age.

Moreover, results of our secondary analyses directly comparing model-associated HbO responses in older and younger adults (Peng et al., 2023) revealed age-related differences in the contributions of the subprocesses of valuation and their neural correlates. The HbO responses in the frontoparietal channels (in the left DLPFC and left SPG) correlated negatively with the process of expectation violation (seeFig. 3B) in older adults. Of note, those older adults with greater such expectation violation-related brain responses relative to those associated with belief updating showed greater deviations from the Bayesian model predictions in their updating behavior (Fig. 3E). In contrast, valuation processes in younger adults were observed to be more associated with belief and value updating, instead of expectation violation (Peng et al., 2023). A direct comparison with data of older adults also showed that value-updating-related activity in the left DLFPC was only significant in younger but not in older adults (Fig. 5B). Together, these results revealed age differences in subprocesses of value-based decisions under different types of uncertainty. Relative to younger adults, the behavior of older adults was primarily associated with expectation violation and its brain substrate. These findings are partly in line with previous studies showing age-related differences in the frontoparietal functional architecture (e.g.,Schlesinger et al., 2017;Setton et al., 2023) as well as with age-related differences in the relative roles of model-free and model-based decision processes (e.g., seeEppinger et al., 2025for a review).

As expected, we observed that older adults updated their values based on whether a draw matched or mismatched the winning color of a gamble, but did not account for whether the draw indicated a change in winning probability and thereby reduced uncertainty (Fig. 2A). Older adults showed significant value updating in all trial categories, irrespective of whether value updating would be normative or non-normative. Specifically, based on the predictions of the Bayesian model, however, normative value updating is only expected in ambiguous gambles with ambiguous color draws where the uncertainty of the winning probability is reduced (i.e., two normative categories). Our results showed that in these trials, value updating is relatively preserved in old age. They did update in these trial types quasi-normatively, deviating from model predictions only in the degree (less updating than expected), but not direction, of updating (Fig. 2B). In contrast, value updating in the remaining three categories is non-normative, since new information at a given draw could not reduce the uncertainty about the winning probability associated with a gamble. Older adults, nevertheless, showed non-normative updating behavior and significantly update their subjective values of the gambles at the postdraw phase in all non-normative categories.

This non-normative updating behavior in older adults differed considerably from earlier findings observed in younger adults. Specifically, results from a secondary analysis that incorporated data from younger adults (Peng et al., 2023) showed that while there was no performance difference between older and younger adults in normative trials, impairments in updating behavior in older adults were apparent in non-normative trial categories (Fig. 4). That is, younger adults did not update subjective values in non-normative trials as predicted by the Bayesian model, whereas older adults updated their subjective values even in situations where uncertainty could not be reduced by new information (see right panel ofFig. 4Afor direct age comparison in value updating). Thus, the results indicate that older adults did not differentiate between situations in which new information did or did not change the winning probability, reflecting insensitivity to the type of uncertainty.

What could be driving older adults’ updating behavior then? Results from fitting Bayesian quantitative predictions to their performance shed light on this question. As shown inTable 2, older adults’ updating behavior was mainly associated with model-based estimates of expectancy violation in the different trial categories. This stands in contrast to earlier findings showing that younger adults’ value updating is mainly driven by belief updating (Peng et al., 2023). It appears that in our task, older adults relied much more on rudimentary, low-level feedback about match or mismatch between draw and winning color, with the strength of positive and negative updating driven by expectancy violation, instead of forming and updating a mental model of the urn composition with new information available in the observed draws. These results may suggest that older adults employ a simpler decision-making strategy, characterized by a reliance on immediate, sensory salient signals, which is consistent with older adults’ increased reliance on intuitive versus deliberative processing (Mikels et al., 2013). Similarly, another study on age-related impairment in sequential decision making (Eppinger et al., 2013) also found that when value-based learning depended on delayed outcomes (i.e., cognitively more demanding model-based reinforcement learning), older adults still focused on the expectancy violation based on immediate outcomes (i.e., relying on the less demanding model-free reinforcement learning). In many theoretical frameworks of decision making, such as model-free reinforcement learning and the Pearce–Hall model, the extent to which new observations (or feedback) violate expectations is informative and is the key process in driving subjective value updating for actions or options in reward-based model-free learning (Pearce & Bouton, 2001;Pearce & Hall, 1980;Roesch et al., 2012;Sutton & Barto, 1998). However, in the natural environment, not all expectancy violations are informative. They might be important for sensory encoding (Tang et al., 2023) but not always systematically inform about the statistic properties of the environment (Kobayashi & Hsu, 2017). Also, new information about the environment might not always resolve uncertainty. For instance, in the case of our gamble bidding task, while an observed draw of ambiguous colors in risky gambles could provide new information about the urn’s contents, it is irrelevant to the calculation of winning probabilities in such situations.

Results based on individual differences at the behavioral and brain levels shed further light on this finding. Individuals with better logical reasoning ability (as measured by the Raven’s test scores) showed less deviations from the model predictions in value updating (Fig. 2C). At the cortical level, we observed correlates of belief updating and expectancy violation in the SPG and the left DLPFC. In line with results from previous fMRI (Kobayashi & Hsu, 2017) and fNIRS (Peng et al., 2023) studies, cortical correlates of these two processes overlapped to a certain extent (Fig. 3A, B); note, however, these process-associated cortical activities correlated with updating performance in different directions, that is, positive for model-based coefficients of belief updating: (mostly) negative for expectancy violation. Importantly, participants with more positive expectancy violation-related HbO activation in the left DLPFC deviated more from Bayesian predictions (Fig. 3D, E). Of note, while HbO activities in this left DLPFC channel was significantly related to expectancy violation, at a liberal threshold (uncorrected*p*< 0.05), this activity also reflected individual differences in belief updating. This differs from previous studies of younger adults which show distinct activities in specific regions. For instance, the same SPG channel was uniquely associated with expectancy violation [*β*= 2.77, FDR*q*= 0.01] but not belief updating [*β*= -0.35, FDR*q*= 0.76] in younger adults (Peng et al., 2023). These findings suggest diminished neural specialization for these processes in older adults, which is in line with the neural dedifferentiation theory (Koen & Rugg, 2019;Koen et al., 2020). According to this theory, as individuals age, distinct brain regions gradually lose their specialized functions, leading to decreased efficiency in processing specific types of cognitive information. This loss of specialization can significantly impact the brain’s ability to manage complex tasks, leading to a general decline in the precision with which information is processed. It seems plausible that neural dedifferentiation in frontoparietal areas such as the DLPFC might be one of the neurobiological mechanisms associated with the diminished sensitivity to uncertainty reduction in older adults.

The DLPFC plays a crucial role in belief updating in younger adults following new evidence, as shown by neuroimaging (Kobayashi et al., 2021), but also by using brain stimulation to test for the causal role of the DLPFC in this process (e.g.,Ambrus et al., 2020;Schulreich & Schwabe, 2021). A recent study found that after applying anodal tDCS over the DLPFC, younger adults showed increased non-normative value updating, potentially driven by expectancy violation (Schulreich & Schwabe, 2021). This pattern is similar to the updating behavior of older adults in our study. Our finding of expectancy violation-related HbO activities in the DLPFC might suggest that older participants interpret the low-level rudimentary information of color match or mismatch without fully considering the statistical contexts of risky and ambiguous gambles as indicative of changes in the decision-making environment, leading to suboptimal updating performance. Consistent with this, we found that older adults with more pronounced expectancy violation HbO activities deviated more from Bayesian predictions (Fig. 3D, E).

Another interesting aspect of the results is that we observed valence-related asymmetry in value updating among older adults, where negative information led to greater value updating than positive information. Our secondary analysis showed that this valence asymmetry was observed both in older and younger adults without significant age differences. This finding is in line with some previous studies suggesting preserved valence effects during value updating across age groups (e.g.,Spaniol & Wegier, 2012). However, other studies on aging and reward-related learning or decision making that used a range of different experimental paradigms showed mixed findings—some found reduced valence asymmetry effects in older adults (Hämmerer et al., 2011;Simon et al., 2010), while some showed older adults learned more from loss (or negative feedbacks) compared with younger adults (e.g.,Freund & Keil, 2021). In our previously published study of younger adults, the valence asymmetry was only observed in normative but not in non-normative trials (Peng et al., 2023). The fact that the valence effect in older adults does not interact with the normativity of the contexts of updating may be due to their insensitivity to uncertainty reduction as discussed above, since older adults updated values both in normative and non-normative contexts, unlike younger adults. These mixed patterns of results in the literature underscore the complex nature of valence-dependent processes in aging and suggest that task-specific contexts may influence how valence effects manifest (Mata et al., 2011). The task we used is a gambling/bidding task that did not have immediate feedback or reward directly associated with each decision (only in the final resolution). This allowed us to isolate the effects of valence asymmetry without the influence of reward amounts (e.g., in reinforcement learning tasks, negative and positive feedback are associated with losing or gaining rewards, respectively).

## 5 Limitations and Future Directions

The current study has advanced our understanding of age-related differences in adaptive behavior under uncertainty. However, due to the limited measurement depth of fNIRS (Cui et al., 2011) and the specific brain areas covered by our montage, we were unable to assess other regions that are also crucial for updating processes. For instance, the striatum, known to encode model-based prediction error signals (Behrens et al., 2008;Daw et al., 2011), aligns conceptually with the value updating observed in our study. We observed only weak HbO activities related to value updating in three prefrontal cortex channels (uncorrected*p*< 0.05,Fig. 3C). It remains unclear whether older adults might employ other regions to encode value updating. To gain a more comprehensive understanding, future research could employ functional magnetic resonance imaging to better assess activities in the midbrain and other subcortical regions. Moreover, due to the temporal resolution limitations of fNIRS, our study could not explore the temporal dynamics of information processing in older adults. Future studies should consider using electroencephalography or magnetoencephalography, which offer higher temporal resolution to capture the rapid neural dynamics involved in processing expectancy violation and resolving uncertainty signals. Although valence asymmetry is not the main focus of this study, future studies on how aging may affect valence-dependent processing could extend our findings by incorporating additional elements, such as trust-based interactions, to examine how valence effects operate in more complex decision-making contexts. For example, studies investigating trust-based decisions have found that younger adults often show heightened sensitivity to negative information (Tzieropoulos, 2013), though similar research in older populations remains scarce (Fareri et al., 2022;Seaman et al., 2023).

## 6 Conclusions

Our study shows that older adults’ updating performance was only relatively preserved when the received information is new and indeed indicated changes in the winning probability of the gambles. However, they exhibit performance impairments in non-normative trials where uncertainty is irreducible or new information is irrelevant to the decision at hand. Their value updating was primarily driven by how much the draw violated their expectancy, which, in the context of our task, is not an informative signal. The individuals whose updating behavior deviates more from the Bayesian model predictions exhibited dedifferentiation of the neural correlates of belief updating and expectancy violation, with updating behavior driven more strongly by expectancy violation-related neural activity. Together, the current study advances our understanding of age-related changes in adaptive behavior under uncertainty and underscores the potential impact of neural dedifferentiation.

## Supplementary Material

Supplementary Material

## Data Availability

The anonymized fNIRS and behavioral data that support the findings of this study are not openly available due to the conditions of the ethical approval we obtained for this study, but are available upon request by researchers. Access to data by qualified investigators must comply with the European Union General Data Protection Regulations (GDPR) and all relevant guidelines. The completion of a data transfer agreement signed by an institutional official will be required. For data access, contact corresponding authors of the study, who will assist the data accessing process and contact the relevant institutional office. The fNIRS analysis code (for Brain AnalyzIR Toolbox in MATLAB), behavioral analysis code, and results are all publicly available at:https://osf.io/yhb2f/
