# Supplementary material for: Neural correlates of reduced sensitivity to information about uncertainty during valuation in older adults: An fNIRS study
Source: Imaging Neurosci (Camb). 2025 Jun 24;3:IMAG.a.61. doi: 10.1162/IMAG.a.61 (PMC12319833; doi:10.1162/IMAG.a.61)
Supplement: Supplementary Material [file imag.a.61_supp.pdf]

Supplementary Materials

Neural correlates of reduced sensitivity to information about uncertainty during valuation in older adults: an fNIRS study

Text S1: Control analysis of color assignments

To prevent the influence of color preferences, we allocated colors—specifically, blue, red, and yellow—to represent risky and ambiguous colors across all participants. We designed three color assignment schemes: (1) blue as the risky color, with red and yellow as ambiguous colors; (2) red as the risky color, with blue and yellow as ambiguous colors; (3) yellow as the risky color, with blue and red as ambiguous colors. These assignments were predetermined based on participant identification numbers to ensure uniform distribution prior to initiating the study. Among the 46 older adults in the final sample, 17 were assigned to the first scheme, 14 to the second, and 15 to the third. Furthermore, to assess the impact of color assignment, we employed a linear mixed-effects model with color assignment and trial category as fixed effects, mean value updating (i.e., ΔWTSs) as the dependent variable, and participants as random effects. Our findings indicated that neither the effect of color assignment ($F_{1,44} = 0.04$, $p = 0.85$) nor the interaction between color assignment and trial category ($F_{4,176} = 0.61$, $p = 0.66$) was significant.

Text S2: Control analysis of potential order effects in older adults

Treating each gambling scenario as independent is a key aspect of task comprehension. To investigate whether the order of scenarios potentially influences older adults' value updating and if the order interacted with different trial categories, we conducted a linear mixed-effect model analysis. This model included scenario order (1st / 2nd / 3rd), trial category (normatively positive / normatively negative / non-normatively positive / non-normatively negative in risky/ambiguous gamble) and their interaction as predictor variables, with trial-wise ΔWTSs (i.e., value updating) as the dependent variable and participants as random effects. ΔWTSs was sign-flipped in the negative trial categories. The result revealed no significant main effect of draw order ($F_{1,3258.7} = 1.16$, $p = .28$, partial $\eta^2 < 0.001$). The interaction effect between draw order and trial category was not significant ($F_{4,3272} = 1.13$, $p = 0.34$, partial $\eta^2 = 0.001$), implying that older adults value updating behavior in different trial categories appears to be unaffected by the order of scenarios.

Text S3: Older adults deviated from the model prediction both in predraw and in postdraw

The predraw valuation performance was quantified by subtracting model predicted predraw values from predraw WTSs of the risky/ambiguous gambles separately. These differences were averaged for each participant and tested against a null hypothesis of zero.

We found that the difference values were significantly greater than zero in both risky (*Mdn* = 0.83, $p < 0.001$, $r_{rb} = 0.71$) and ambiguous gambles (*Mdn* = 1.90, $p < 0.001$, $r_{rb} = 0.82$), showing that the predraw subjective values of the gambles in older adults was higher than the Bayesian model predictions. Furthermore, the difference values of ambiguous gambles were significantly larger than the risky gambles (paired-wise Wilcoxon signed rank test $p = 0.002$).

Participants' postdraw WTSs were also greater than prediction in the normatively positive trials (*Mdn* = 0.88, $p < 0.001$, $r_{rb} = 0.62$), normatively negative trials (*Mdn* = 2.13, $p < 0.001$, $r_{rb} = 0.86$), non-normatively positive trials (*Mdn* = 1.04, $p < 0.001$, $r_{rb} = 0.78$), and non-normatively negative trials in ambiguous gambles (*Mdn* = 1.67, $p < 0.001$, $r_{rb} = 0.79$). The only category that did not significantly differ from the model predictions was the non-normatively negative trials in risky gambles (*Mdn* = -0.15, $p = 0.19$, $r_{rb} = 0.19$). These results indicate that even though older adults ascribed higher values to the gambles than the model predictions at the predraw phase, the higher predraw values did not limit them from giving greater values than the model predictions at the postdraw phase.

Table S1 *Urn content compositions with respective predictions of belief updating, value updating and expectancy violation under the Bayesian model for different draws*

Urn Contents			Ambiguous-color draws				Risky-color draw			
Urn ID	n of Balls in Ambiguous color (EV[1])	n of Balls in risky color (EV[1])	Belief updating	Value updating: Ambiguous gamble	Value updating: Risky gamble	Expectancy violation	Belief updating	Value updating: Ambiguous gamble	Value updating: Risky gamble	Expectancy violation
**1**	1 (2.50)	1 (5.00)	0.250	±2.50	0	0.750	0	0	0	0.500
**2**	1 (1.67)	2 (6.67)	0.167	±1.67	0	0.833	0	0	0	0.333
**3**	2 (3.33)	1 (3.33)	0.167	±1.67	0	0.667	0	0	0	0.667
**4**	1 (1.25)	3 (7.50)	0.125	±1.25	0	0.875	0	0	0	0.250
**5**	2 (2.50)	2 (5.00)	0.125	±1.25	0	0.750	0	0	0	0.500
**6**	3 (3.75)	1 (2.50)	0.125	±1.25	0	0.625	0	0	0	0.750

Note. [1] Values shown in the parathesis are model-based predictions of initial (predraw) expected values (in Euros) of the gambles for which the colors of the balls drawn match the winning colors.

Table S2 *Instruction quiz questions and accuracy based on the participants' initial answers (translated English version of the task instruction slides are included in the Appendix section below)*

No	Questions	Correct answer	Accuracy YA	Accuracy OA	YA vs. OA χ^2 (p)
1	How many balls in the urn are purple? 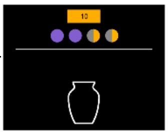	Two	97.78 %	95.65 %	0 (1)
2	How many balls in the urn are gray?	It is not possible to know for sure how many are grey.	95.56 %	84.78 %	1.88 (0.17)
3	How many orange balls are still in the urn in this Scenario? 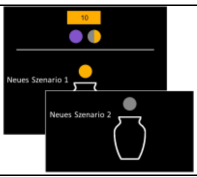	No more orange balls in the urn.	88.89 %	89.13 %	0 (1)
4.1	Suppose the randomly selected lottery at the end is one that you gave a value of € 3, and the computer-generated random price for this lottery is € 4. Could you play the lottery according to the rule of the game?	No, because the value I gave (€3) is lower than the computer generated price (€4).	91.11 %	84.78 %	0.37 (0.54)
4.2	How much bonus money would you receive in the case of Q4.1?	You receive €4 by selling the lottery at the price of the computer-generated value.	64.44 %	60.87 %	0.02 (0.89)
5.1	Suppose the randomly selected lottery at the end is one that you gave a value of € 5, and the computer-generated random price for this lottery is € 4. Could you play the lottery according to the rule of the game?	Yes, because the value I gave (€5) is higher than the computer-generated price (€4).	95.56 %	95.65 %	0 (1)
5.2	How much bonus money would you receive in the case of Q5.1?	I have a chance to win €10 if the color of the drawn ball matches the winning color; otherwise, I would win nothing.	71.11 %	71.74 %	0 (1)
6	The winning color as shown here is orange. An orange ball was drawn in Scenario 1. The value you entered for this lottery is higher than the computer-generated price. Therefore, you are allowed to play the lottery at the very end of the task. In the final drawing, a purple ball is drawn. How much money would you receive? 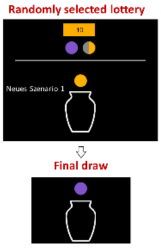	Receive nothing because the color of the ball drawn in the final draw is purple, which does not match the winning color which is orange.	91.11 %	89.13 %	0 (1)

Note. After reading through the instruction slides (see *translated English version of the task instruction slides are included in the Appendix section below*), all participants completed the above quiz with 8 questions administered by the experimenter. When participants answered incorrectly, the experimenter immediately referred them back to the relevant instruction section and provided clarification before moving to the next question. Results of the analyses

shown in the table were based on the participants' initial answers before the experimenter provided further explanations. The results show that older adults and younger adults had similar initial accuracy for each of the questions, suggesting comparable basic understanding of the task between age groups before the practice (mean accuracy for older adults = 0.82, *SD* = 0.23; mean accuracy for younger adults = 0.85, *SD* = 0.16; $t_{89} = -0.73$, $p = 0.46$).

Table S3 Age comparisons based on secondary analysis. Best-fitting models on the influence of age group (old vs. young), belief updating, and expectancy violation on value updating in different trial categories.

	Coefficient	SE	DF	95% CI	*p*-value
Normatively positive (ambiguous-color draws in ambiguous gambles—color matched)					
**(Intercept)**	**-1.63**	**0.54**	**505.19**	**[-2.69, -0.58]**	**0.002**
**Belief updating**	**3.78**	**1.25**	**455.00**	**[1.32, 6.23]**	**0.003**
**Expectancy violation**	**2.33**	**0.64**	**455.00**	**[1.07, 3.59]**	**< 0.001**
Normatively negative (ambiguous-color draws in ambiguous gambles—color mismatched)					
**(Intercept)**	**0.84**	**0.32**	**513.36**	**[0.21, 1.48]**	**0.010**
**Age**	**-1.56**	**0.46**	**513.63**	**[-2.45, -0.66]**	**< 0.001**
**Belief updating**	**-13.83**	**1.74**	**455.00**	**[-17.25, -10.40]**	**< 0.001**
**Age × Belief updating**	**10.49**	**2.45**	**455.00**	**[5.67, 15.30]**	**< 0.001**
Non-normatively positive (risky-color draws in risky gambles)					
**(Intercept)**	**-0.68**	**0.24**	**401.55**	**[-1.14, -0.21]**	**0.005**
**Age**	**0.81**	**0.21**	**91.20**	**[0.39, 1.22]**	**< 0.001**
**Expectancy violation**	**0.75**	**0.37**	**453.60**	**[0.02, 1.48]**	**0.043**
Non-normatively negative (risky-color draws in ambiguous gambles)					
(Intercept)	1.41	0.88	494.35	[-0.32, 3.13]	0.109
Age	2.12	1.24	494.36	[-0.31, 4.55]	0.087
Expectancy violation	-2.13	1.14	454.01	[-4.38, 0.11]	0.062
**Age × Expectancy violation**	**-4.12**	**1.61**	**454.20**	**[-7.28, -0.96]**	**0.011**
Non-normatively negative (ambiguous-color draws in risky gambles)					
(Intercept)	0.43	0.36	545.98	[-0.28, 1.13]	0.237
**Age**	**1.17**	**0.51**	**545.98**	**[0.17, 2.16]**	**0.022**
**Expectancy violation**	**-1.34**	**0.41**	**455.00**	**[-2.15, -0.53]**	**0.001**
Belief updating	0.67	1.61	455.00	[-2.49, 3.83]	0.676
**Age × Expectancy violation**	**-1.56**	**0.58**	**455.00**	**[-2.70, -0.42]**	**0.007**
**Age × Belief updating**	**-4.87**	**2.26**	**455.00**	**[-9.31, -0.43]**	**0.032**

Note. Bold font indicates statistically significant result ($p < 0.05$). Maximum-likelihood mixed-effect models of all candidate variables were created using the *lme4::lmer* function. The *lmerTest::step* function was used to back-fit maximum-likelihood models for all candidate variables, with a significance cut-off of 0.05 for retaining fixed effects.

Table S4 Estimation of linear mixed-effect models on the influence of belief updating, and expectancy violation on value updating in different trial categories with Raven's test scores and processing speed for each participant as covariates in the models.

	Coefficients (βs)	SE	DF	95% CI	*p*-value
Normatively positive (ambiguous-color draws in ambiguous gambles—color matched)					
(Intercept)	-0.008	1.84	66.59	[-3.67, 3.66]	0.996
Belief updating	1.84	1.93	220.00	[-1.97, 5.64]	0.343
Expectancy violation	1.77	0.99	220.00	[-0.18, 3.72]	0.075
Raven scores	-0.02	0.04	44.00	[-0.09, 0.05]	0.587
Normatively negative (ambiguous-color draws in ambiguous gambles—color mismatched)					
(Intercept)	-1.63	1.57	47.69	[-4.78, 1.51]	0.302
**Belief updating**	**-3.94**	**1.95**	**220.00**	**[-7.79, -0.09]**	**0.045**
Raven scores	0.02	0.03	44.00	[-0.05, 0.09]	0.509
Non-normatively positive (risky-color draws in risky gambles)					
(Intercept)	1.00	1.22	50.22	[-1.44, 3.45]	0.414
Expectancy violation	0.86	0.62	219.50	[-0.36, 2.07]	0.167
Raven scores	-0.02	0.03	44.01	[-0.07, 0.03]	0.414
Non-normatively negative (risky-color draws in ambiguous gambles)					
(Intercept)	1.96	1.90	90.28	[-1.81, 5.74]	0.304
**Expectancy violation**	**-5.82**	**1.43**	**219.26**	**[-8.64, -3.01]**	**< 0.001**
Raven scores	0.03	0.03	43.88	[-0.04, 0.10]	0.408
Non-normatively negative (ambiguous-color draws in risky gambles)					
(Intercept)	0.36	1.41	52.00	[-2.46, 3.18]	0.797
**Expectancy violation**	**-2.87**	**0.50**	**220.00**	**[-3.86, -1.88]**	**< 0.001**
**Belief updating**	**-4.15**	**1.95**	**220.00**	**[-8.00, -0.30]**	**0.035**
Raven scores	0.03	0.03	44.00	[-0.03, 0.09]	0.370

Note. Due to invalid Raven scores (multiple answer selections or excessive non-responses), two participants were excluded from this covariation analyses (simple size here = 44). Value updating (i.e., ΔWTS) in normatively/non-normatively negative trials was not sign-flipped here. Bold font indicates statistically significant result ($p < 0.05$).

Table S5 Number of older adults with good signal quality for each channel.

Channel	Source No.	Source	Detector No.	Detector	Number of participants with good signal quality
1	1	AF8	2	Fp2	40
2	1	AF8	4	F6	41
3	2	AF4	2	Fp2	43
4	2	AF4	4	F6	43
5	2	AF4	1	AFz	42
6	3	F4	3	F2	41
7	3	F4	4	F6	44
8	3	F4	5	FC4	42
9	4	FC2	3	F2	34
10	4	FC2	5	FC4	36
11	4	FC2	6	C2	36
12	5	C4	5	FC4	41
13	5	C4	6	C2	46
14	5	C4	7	CP4	45
15	6	CP2	6	C2	45
16	6	CP2	7	CP4	46
17	6	CP2	8	P2	44
18	7	CP6	7	CP4	45
19	8	P4	7	CP4	45
20	8	P4	8	P2	44
21	14	CP1	15	CP3	45
22	14	CP1	16	P1	45
23	14	CP1	14	C1	45
24	14	CP1	9	CPz	46
25	15	CP5	15	CP3	46
26	16	P3	15	CP3	46
27	16	P3	16	P1	46
28	13	C3	15	CP3	46
29	13	C3	14	C1	46
30	13	C3	13	FC3	46
31	12	FC1	14	C1	45
32	12	FC1	13	FC3	41
33	12	FC1	11	F1	41
34	11	F3	13	FC3	45
35	11	F3	11	F1	43
36	11	F3	12	F5	46
37	9	AF7	10	Fp1	46
38	9	AF7	12	F5	46
39	10	AF3	10	Fp1	41
40	10	AF3	12	F5	41

Note. The summaries were based on the included 46 older adult.

Table S6 *Effects of valence and normativity on frontoparietal activity during scenario presentations in older adults*

Channel	S	D	AAL Label	*t*-stat	*FDR q*
HbO:					
Main effects of normativity					
No significant channels were identified					
Main effects of valence					
10	4	5	Frontal_Mid_2_R	4.166	0.001
17	6	8	Parietal_Sup_R	3.343	0.011
39	10	10	Frontal_Sup_2_L	3.844	0.002
32	12	13	Frontal_Mid_2_L	4.019	0.001
33	12	11	Frontal_Sup_2_L	2.852	0.034
28	13	15	Parietal_Inf_L	2.882	0.034
23	14	14	Postcentral_L	3.192	0.016
22	14	16	Parietal_Sup_L	2.871	0.034
Normativity × valence interactions					
No significant channels were identified					
HbR:					
Main effects of normativity					
No significant channels were identified					
Main effects of valence					
32	12	13	Frontal_Mid_2_L	1.143	0.001
Normativity × valence interactions					
No significant channels were identified					

Note. Significant channels identified as FDR-corrected $q < 0.05$. AAL labels defined by maximal coverage of talairach daemon parcellation ROI.

Table S7 *Effects of updating categories on frontoparietal activation during scenario presentations in older adults*

Channel	S	D	Label AAL	*beta*	*t*-stat	*FDR q*
HbO:						
Normatively positive vs. normatively negative						
11	4	6	Frontal_Sup_2_R	-3.747	-4.904	< 0.001
10	4	5	Frontal_Mid_2_R	-3.372	-3.607	0.010
17	6	8	Parietal_Sup_R	-3.315	-4.005	0.002
32	12	13	Frontal_Mid_2_L	-3.259	-4.170	0.001
Normatively positive vs. normatively zero						
3	2	2	Frontal_Sup_2_R	2.452	3.294	0.024
11	4	6	Frontal_Sup_2_R	-3.018	-4.753	< 0.001
10	4	5	Frontal_Mid_2_R	-3.586	-4.620	< 0.001
17	6	8	Parietal_Sup_R	-2.971	-4.340	0.001
23	14	14	Postcentral_L	-1.904	-3.096	0.043
Normatively negative vs. normatively zero						
32	12	13	Frontal_Mid_2_L	1.944	3.074	0.043
HbR:						
Normatively positive vs. normatively negative						
No significant channels were identified						
Normatively positive vs. normatively zero						
1	1	2	Frontal_Mid_2_R	0.647	3.354	0.022
Normatively negative vs. normatively zero						
No significant channels were identified						

Note. Significant channels identified as FDR-corrected $q < 0.05$. AAL labels defined by maximal coverage of talairach daemon parcellation ROI.

Table S8 *HbO Activation of channels significantly associated with belief updating, expectancy violation and value updating during scenario presentations in older adults*

Channel	S	D	AAL Label	beta	*t*-stat	*FDR q*
Parametric modulator: model predicted belief updating						
23	14	14	Postcentral_L<br>Parietal_Sup_L	2.281	4.456	0.002
Parametric modulator: model predicted expectancy violation						
23	14	14	Postcentral_L<br>Parietal_Sup_L	-2.543	-3.511	0.041
34	11	13	Frontal_Mid_2_L	-3.718	-4.350	0.002
Parametric modulator: observed value updating						
No significant channels were identified						

Note. Significant channels identified as FDR-corrected $q < 0.05$. AAL labels defined by coverage of talairach daemon parcellation ROI. We also conducted analyses of HbR, and no channels showed significant activity related to any of the updating processes (uncorrected *p*s > 0.07).

Appendix: English translations of the instruction slides of the gambling bidding task. The instructions were given in a one-on-one interactive manner. Each participant read the instruction on each slide with an experimenter next him/her and can ask questions about any parts of the instructions. The experimenter provided explanations for the questions

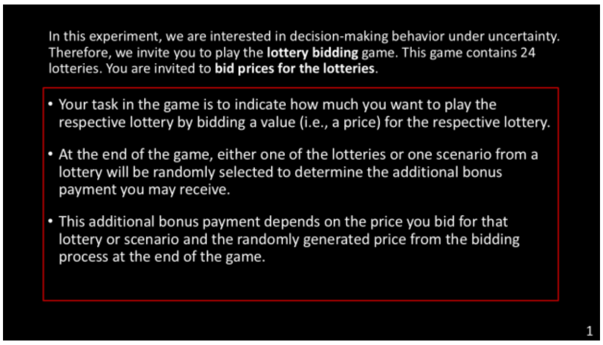

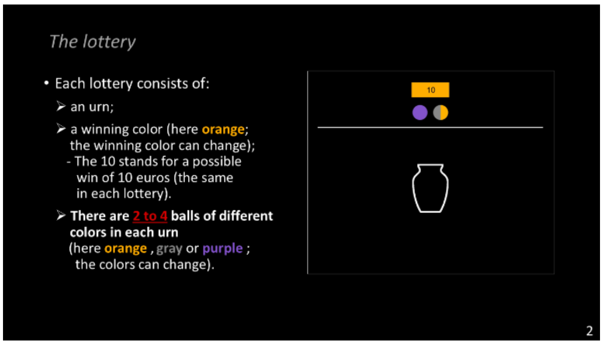

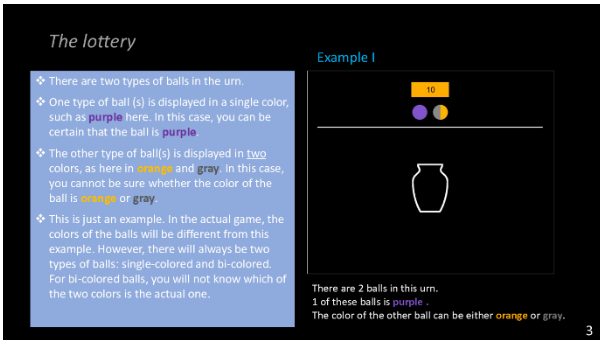

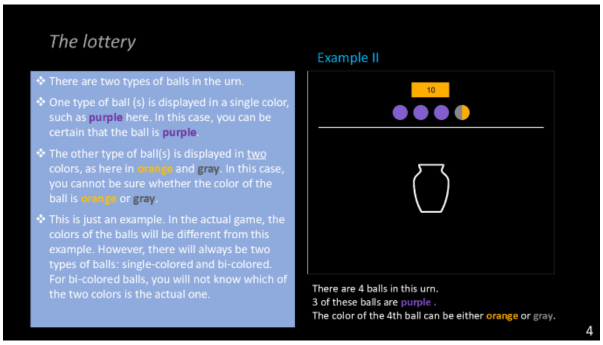

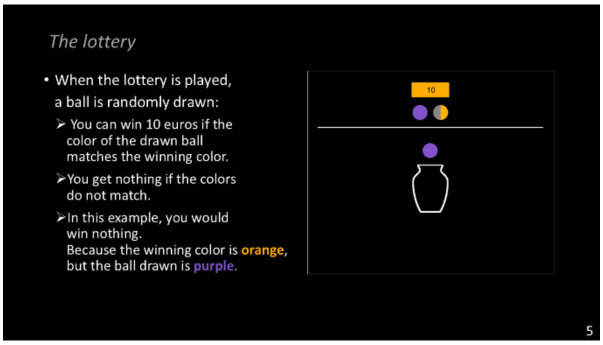

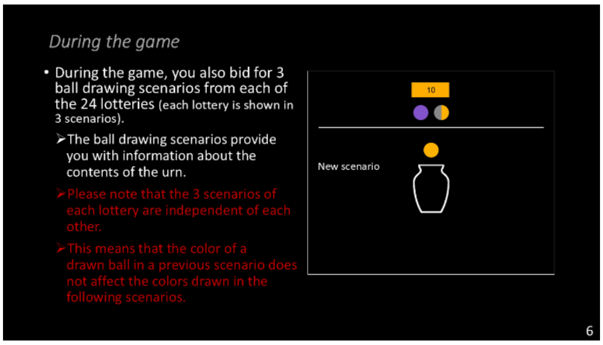

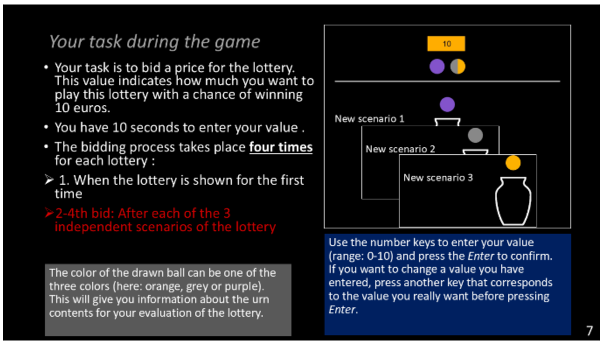

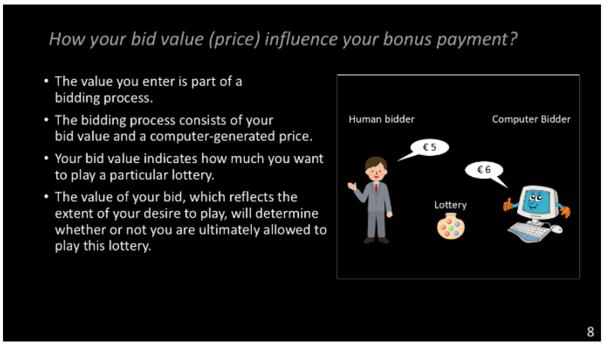

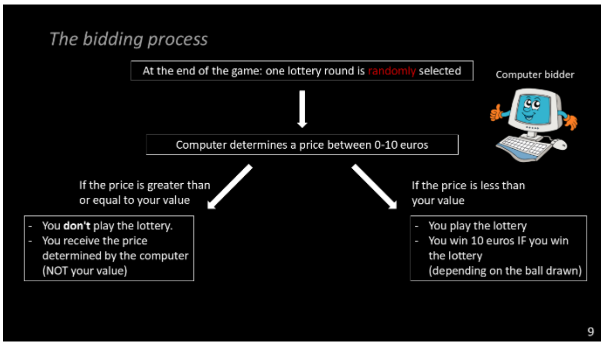

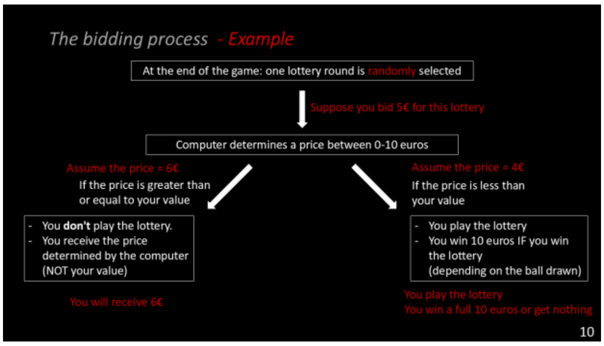

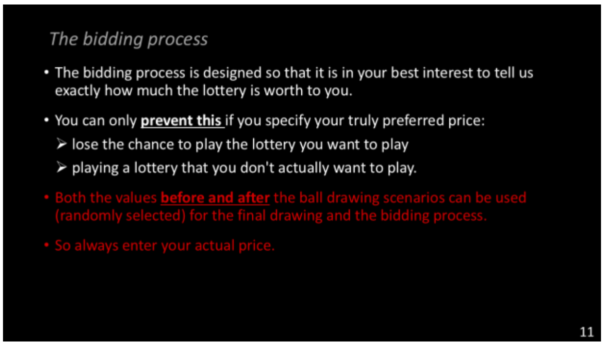

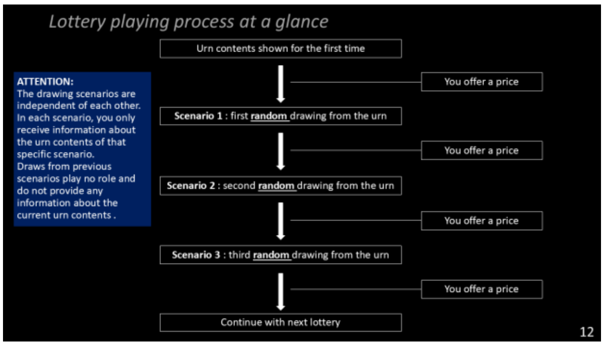

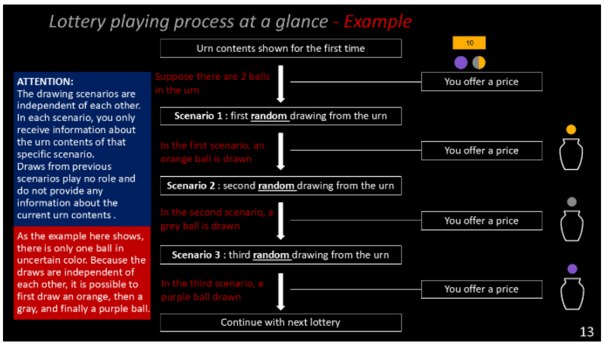

Note. Translated into English. These slides were presented to participants using progressive reveal animation, where each point appeared sequentially.
